# Polyphenol-polysaccharide interactions: molecular mechanisms and potential applications in food systems – a comprehensive review

**DOI:** 10.1186/s43014-025-00322-3

**Published:** 2025-08-29

**Authors:** Fereidoon Shahidi, Kerthika Devi Athiyappan

**Affiliations:** Department of Biochemistry, Memorial University of Newfoundland, St. John’s, NL A1C 5S7 Canada

**Keywords:** Polyphenols, Polysaccharides, Covalent interactions, Non-covalent interactions, Polyphenol-polysaccharide complex

## Abstract

Polyphenols, a major class of plant secondary metabolites, are well known for their diverse bioactive properties. It has also been established that polyphenols interact with other macromolecules, such as proteins, polysaccharides, and lipids in the food matrix. Among the primary metabolites of the plant, carbohydrates play a significant role. Polyphenols and polysaccharides form complexes upon interaction; this interaction could be through covalent or non-covalent bonds, such as electrostatic, hydrophobic, van der Waals forces, and hydrogen bonding. These polysaccharide–polyphenol complexes exhibit enhanced bioactivity and influence the digestibility of complex macronutrients (such as proteins and polysaccharides), as well as their biological efficacy, bioavailability, and stability. Despite their numerous benefits and potential applications, the underlying mechanisms of interaction and complex formation between polysaccharides and polyphenols, as well as the influence of their structural parameters, remain underexplored. This comprehensive review summarizes the basic molecular-level implications of polysaccharides and polyphenols, exploring their potential applications in the food industry, and provides a basic understanding of their occurrence in various food matrices, characteristics of both polyphenols and polysaccharides that influence their interaction mechanisms, and detection under laboratory conditions. The review aims to bridge the gap between the molecular-level understanding of the complex and the development of potential nutraceuticals and functional food ingredients based on polysaccharide-polyphenol complexation.

**Graphical Abstract:**

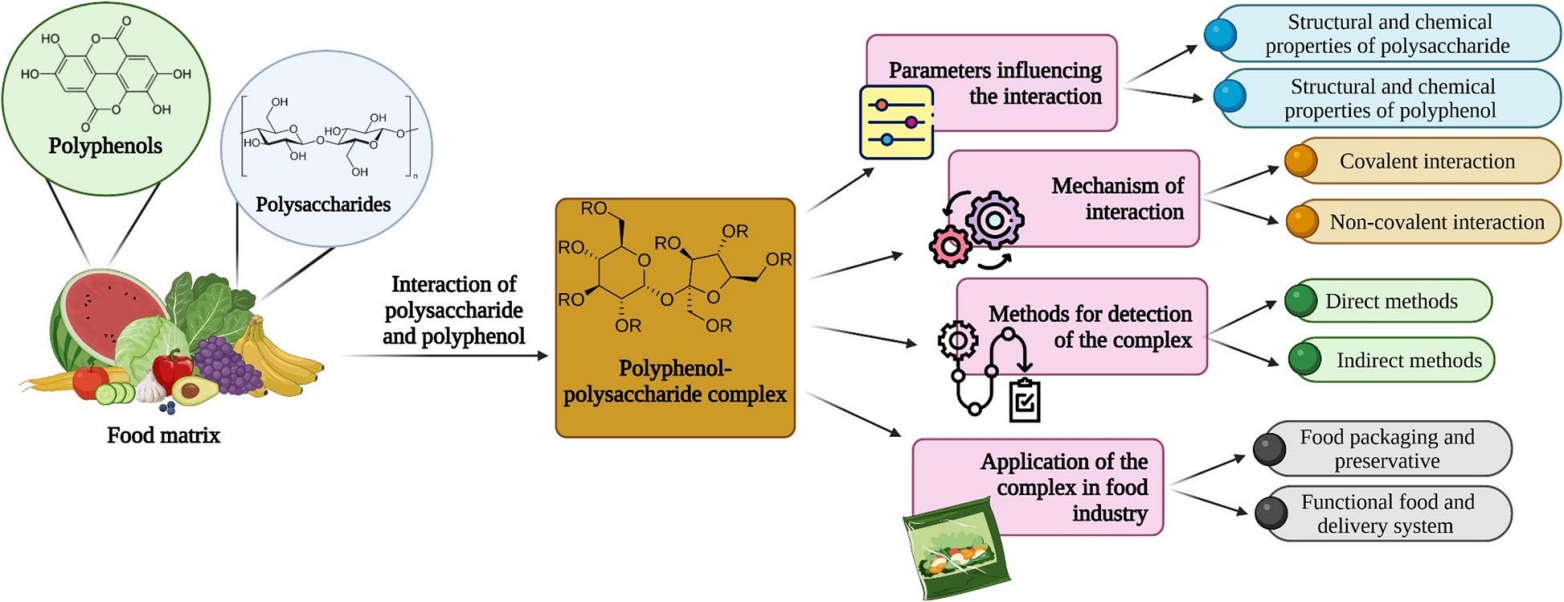

## Introduction

The interaction of polyphenols with macromolecules in the food matrix, such as protein, carbohydrates (specifically polysaccharides), and lipids, has gained much attention from researchers due to the significant influence on the nutritional properties of the food matrix (Das et al., 2020). The interaction can occur covalently (complexation) or non-covalently (conjugation) and might alter the physiological properties of food, including its bioavailability, bioaccessibility, biological efficacy, stability, and digestibility (Eran et al., 2020).

Polyphenols and polysaccharides co-exist in most plant-based foods. In plants, polyphenols are primarily localized in vacuoles, separated from the plant cell wall by cell membranes. Therefore, interaction between the cell wall polysaccharides and polyphenols within the living cells of plants is limited. Some exceptions still exist wherein the polyphenols would be an integral part of the plant cell wall (primary cell wall) (Liu et al., 2020). It was observed that the interaction between polyphenols and polysaccharides occurs within the plant when there is physical damage, and the plant has attained senescence. The bioavailability of the polyphenols and their extractability during food processing depend on the complex formation among polyphenols and other macromolecules. The levels of phenolic acids and procyanidins in processed juices (e.g., apple and pear) and wine are lower than in whole fruit (Bindon et al., 2012).

Some studies have observed that polyphenol oxidase (PPO), particularly active in damaged plant cells, can oxidize polyphenols to form reactive quinones. These oxidized quinones can subsequently interact with polysaccharides, leading to covalent complexation. This oxidation-driven interaction may alter the antioxidant capacity of polyphenols, potentially reducing their bioavailability and functional properties. Furthermore, structural changes resulting from oxidation and complex formation can influence the digestibility of the complexes and their interactions with the gut microbiota (Guyot et al., 2003; Le Bourvellec et al., 2009). There are abundant research articles found on the potential health benefits of the polyphenol-polysaccharide complexes, primarily since this interaction modulates the fermentation of the polyphenols in the gut, whereby impacting their metabolism (Le Bourvellec et al., 2019; Phan et al., 2020; Tarko & Duda-Chodak, 2020). Several articles put forth the mechanism of the interaction of polyphenols with other macromolecules and their impact on the biological system (He et al., 2021; Le Bourvellec et al., 2019; Watrelot et al., 2017; Zhu, 2015). However, very few review articles have found the importance of the impact of parameters of both polysaccharides and polyphenols upon their interaction (Zhu 2018; Liu 2020).

This review briefly focuses on the basic structure of polyphenols and polysaccharides and the parameters influencing their interaction. Additionally, it compiles insights into the mechanisms underlying their interaction and the analytical techniques used to detect these complexes. The application of these complexes in the food industry is also briefly discussed. In a nutshell, this review will provide vital structural information, the mechanism of interaction, analytical methods for detection, and the application of the polyphenol-polysaccharide complexes in different areas of food and beyond.

## Polyphenols in plants: an overview

Phenolic and polyphenolic compounds are major secondary metabolites of plants. They are classified into different families based on the structure of their carbon skeleton and the number and position of each substituent. Over 8,000 molecules of phenols/polyphenols are found in nature. The four main classes of the phenolic family include flavonoids, phenolic acids, lignans, and stilbenes (Cosme et al., 2020; Truzzi et al., 2021). These compounds serve crucial functions in plants, such as pigmentation, which, in turn, enhances pollination by attracting insects such as bees, moths, and flies. Additionally, they also play a pivotal role in protecting plants from microbes, pests, and UV rays (Cianciosi et al., 2022).

**Fig. 1:**
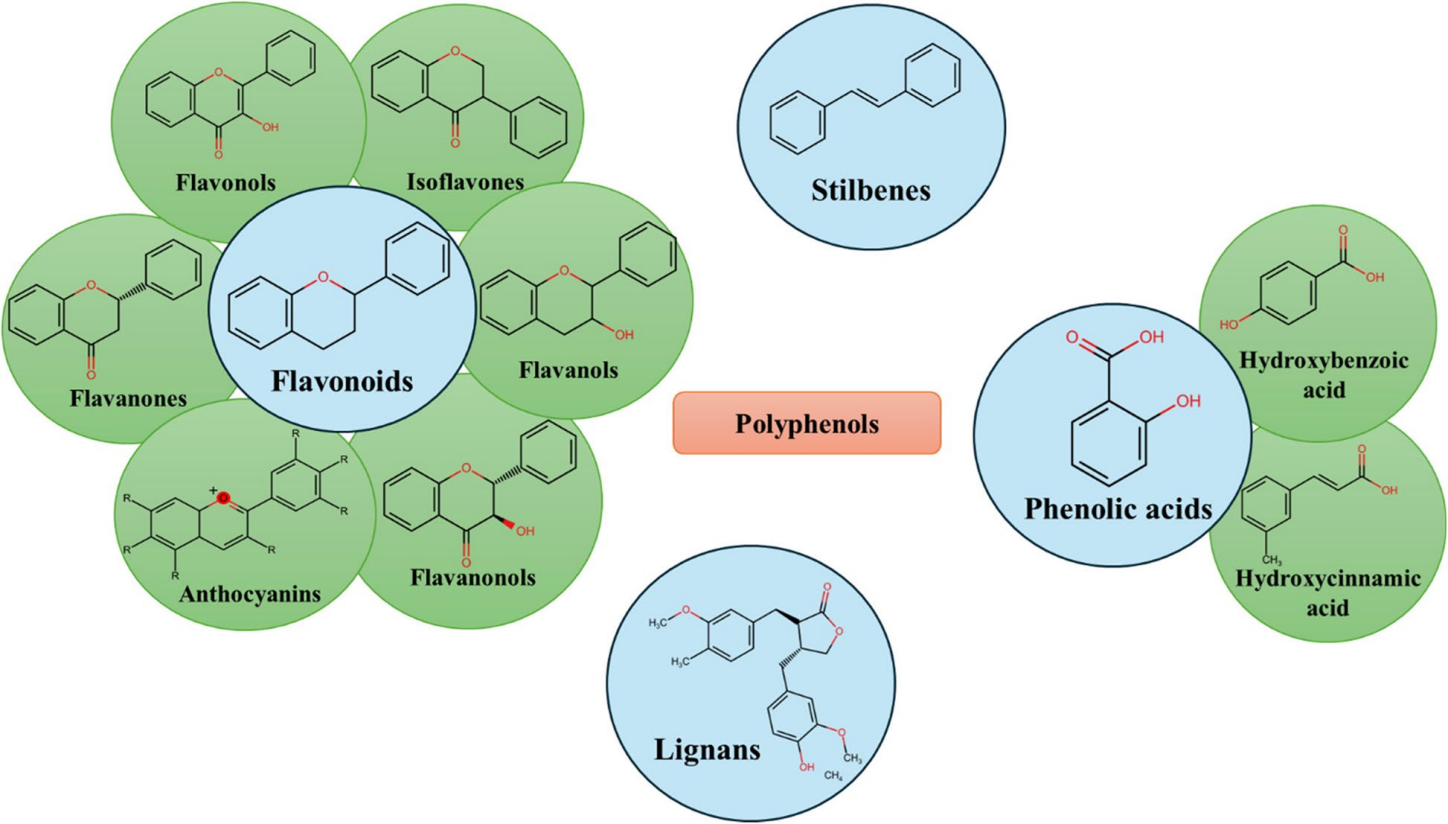
Chemical structures of the major classes and subclasses of phenolic and polyphenolic compounds (Created using Microsoft PowerPoint)

Flavonoids have C6-C3-C6 carbon skeletons, which comprise two benzene rings (rings A and B) connected by a 3-carbon heterocyclic ring (ring C). The flavonoid family is divided into subclasses (Fig. 1) based on the modifications of the three rings (A, B, and C). These modifications include hydroxylation, methylation, methoxylation, and glycosylation of the three rings, different binding possibilities between rings B and C, and diverse structural characteristics of ring B.

Most flavonoids play a significant role in plant development and defence (Cianciosi et al., 2022). Flavonoids comprise some major pigments that impart a wide range of colors in plants, such as anthocyanins, chalcones, flavonols, and flavones (Liu et al., 2021). They possess antioxidant, antimicrobial, anti-diabetic, anti-carcinogenic, and anti-inflammatory properties. By incorporating them into food, their bioactives also enhance the sensory properties, especially the flavor of the products, and serve as a potential functional food ingredient (Shahidi & Dissanayaka, 2023). *Vitis vinifera* L*.*, *Glycine max* L*.*, *Allium cepa* L., and *Citrus sinensis* are some common sources of flavonoids (Table 1).

**Table 1:** Examples of flavonoids present in different plant species

Flavonoid	Example	Common plant species	References
Isoflavones	Genistein	• *Genista tinctoria* L • *Medicago sativa* L • *Lupinus albus* L	Zagórska‐dziok et al. 2021; Hou et al., 2021; Garbiec et al., 2022
	Daidzein	• *Phoenix dactylifera* L • *Vitis vinifera* L • *Ribes. Nigrum* • *Ribes rubrum* • *Glycine max* L	Liggins et al., 2000; Ubaid et al., 2023
	Formononetin	• *Trifolium pratense* • *Cicer arietinum* • *Spatholobus suberectus* • *Glycyrrhiza glabra*	Butkutė et al., 2012; Benedec et al., 2012
	Glycitein	• *Glycine max* L • *Phaseolus vulgaris*	Danciu et al., 2018; Horitani et al., 2024
Flavones	Apigenin	• *Chamaemelum nobile* • *Matricaria chamomilla* • *Trigonella foenum-graecum*	Salehi et al., 2019
	Luteolin	• *Petroselinum crispum* • *Thymus vulgaris* • *Apium graveolens* L • *Olea europaea* L • *Allium cepa* • *Brassica oleracea* • *Malus domestica* • *Daucus carota*	Manzoor et al., 2019; Muruganathan et al., 2022
	Chrysin	• *Passiflora caerulea* • *Momordica charantia* • *Pyrus pashia* • *Banxia Xiexin* • *Diaphragma juglandis*	Coleman et al., 2020; Maasomi et al., 2017; Shahbaz et al., 2023; Zhang et al., 2020
Flavonols	Rutin	• *Fagopyrum sculentum Moench* • *Guiera senegalensis* • *Capparis spinosa* L • *Allium cepa* L • *Asparagus officinalis* L • *Bauhinia fortificata* L	Alam et al., 2017; Frutos et al., 2018; Kočevar Glavač et al., 2017; Motoki et al., 2012; Musallam et al., 2012; Shi et al., 2016
	Quercetin	• *Lactuca sativa* • *Asparagus officinalis* • *Allium cepa* • *Ginkgo biloba* • *Hypericum perforatum* • *Camellia sinensis* • *Sambucus canadensis* • *Brassica oleracea*	Mlcek et al., 2016; Nishimuro et al., 2015
	Kaempferol	• *Capparis spinosa* • *Crocus sativus* • *Allium cepa* • *Brassica oleracea* • *Zingiber officinale* • *Phaseolus vulgaris*	Bhagwat et al., 2013; Periferakis et al., 2022
	Myricetin	• *Brassica napus* • *Vaccinium subg. Oxycoccus* • *Vaccinium sect. Cyanococcus* • *Capsicum frutescens* • *Vicia faba* • Primulaceae	Imran et al., 2021; Semwal et al., 2016
	Isorhamnetin	• *Opuntia ficus-indica* • *Hippophae rhamnoides* • *Ginkgo biloba*	Wang et al., 2023a, 2023b; Wang et al., 2019
Flavanones	Naringenin	• *Vitis vinifera* • *Citrus sinensis* • *Citrus limon* • *Solanum lycopersicum* • *Solanum tuberosum* • *Litchi chinensis* • *Withania somnifera* • *Camellia sinensis*	Alam et al., 2011; Hasegawa et al., 2011; Ma, et al., 2022; Ramesh Prasad Pandey et al., 2015
	Hesperidin	• *Citrus sinensis* • *Citrus reticulata* • *Zanthoxylum avicennae (Lam.) DC* • *Zanthoxylum cuspidatum Champ. ex Benth* • *Acanthopanax setchuenensis Harms ex Diels*	Gómez-Mejía et al., 2019; Pyrzynska, 2022
	Neohesperidin	• *Citrus aurantium* • *Citrus wilsonii Tanaka*	Ortiz et al., 2022
	Eriodictyol	• *Smilax corbularia* • *Citrus sinensis* • *C. aurantium* • *C. reticulata* • *Rhodiola sachalinensis* • *Blumea aromatic*	Guo et al., 2023; Lan et al., 2012
Anthocyanidins	Cyanidin	• *Aronia melanocarpa* • *Prunus domestica* • *Vitis spp.* • *Malus domestica* • *Amelanchier arborea* • *Vaccinium vitis-idaea* L • *Empetrum nigrum* • *Pistacia Vera* L	Andersen & Jordheim, 2010; Han et al., 2019; Liang et al., 2021
	Delphinidin	• *Hibiscus sabdariffa* • *Capsicum annuum* L • *Solanum melongena* L*.’* • *Ribes nigrum* L • *Vaccinium myrtillus* L • *Vaccinium corymbosum* L • *Vaccinium angustifolium*	Chorfa et al., 2016; Dranca & Oroian, 2016; Husain et al., 2022; Ifie et al., 2016; Yao et al., 2015
	Pelargonidin	• *Punica granatum* L • *Fragaria x ananassa Duch* • *Phaseolus spp.* • *Daucus carota* L • *Solanum tuberosum* L • *Raphanus sativus* L • *Glycine max (*L*.) Merr* • *Ipomoea batatas* L • *Brassica campestris* L • *Zea mays* L	Hu et al., 2017; Zhang et al., 2019; Kim et al., 2015; Koh et al., 2014; Oertel et al., 2017
	Peonidin	• *Highbush Blueberry* • *Lowbush Blueberry* • *Cranberry: V. oxycoccus* L • *V. macrocarpon Ait* • *Vitis vinifera* L • *Pyrus spp.* • *Prunus domestica* L • *Phaseolus spp.* • *Zingiber officinale Roscoe* • *Solanum tuberosum* L • *Allium cepa* L • *Ipomoea batatas* L • *Zea mays* L • *Oryza sativa* L • *Syzygium malaccense* (L.)* Merr*	Brahem et al., 2017; da Silva et al., 2017; Zhang et al., 2019; Koh et al., 2014; Li et al., 2012; Oertel et al., 2017
	Petunidin	• *Vaccinium myrtillus* L • *Blueberry: V. corymbosum* L • *V. angustifolium Ait* • *Vitis vinifera* L • *Phaseolus spp.* • *Solanum melongena* L • *Solanum tuberosum* L • *Glycine max* (L.)* Merr* • *Hordeum vulgare* L • *Oryza sativa* L • *Secale cereale* L • *Triticum spp* • *Dovyalis hebecarpa (Gardner) Warb* • *Syzygium cumini (L.) Skeels*	Bellido & Beta, 2009; Chen et al., 2012; de Rosso & Mercadante, 2007; Hu et al., 2017
	Malvidin	• *Morus spp.* • *Berberis lycium Royle* • *Vaccinium myrtillus* L • *Vaccinium corymbosum* L • *Canarium odonthophyllum Miq* • *Vitis vinifera* L • Red wine • *Prunus avium* L • *Solanum lycopersicum* L	Blando et al., 2019; Chew et al., 2012; Gonçalves et al., 2017, 2021; Kharadze et al., 2018; Kim & Lee, 2020; Lee et al., 2016; Merecz-Sadowska et al., 2023
Flavan-3-ols	Catechin	• *Rubus grandifolius* L • *Paullinia cupana* • *Malus domestica* • *Vaccinium subg. Oxycoccus* • *Persea americana* • Green, black, and white teas	Pedro et al., 2020; Silva et al., 2018; Spínola et al., 2019; Wong et al., 2016
	Epicatechin	• *Vitis vinifera* • *Prunus armeniaca* • *Prunus avium* • *Pyrus communis* • *Malus domestica* • *Theobroma cacao* • *Corynanthe pachyceras*	Kouamé et al., 2020
	Gallocatechin	• *Musa Cavendish* • *Mentha pulegium* • *Musa sapientum Linn* • *Ricinodendron heudelotii (Baill.)*	Hadi et al., 2017; Sut et al., 2020; Tongkaew et al., 2022
	Epigallocatechin	• *Camellia sinensis* • *Ziziphus joazeiro*	Ambigaipalan et al., 2020; de Lima Oliveira et al., 2020

The carbon skeleton of phenols is C6, and that of phenolic acids (hydroxybenzoic acids) is C6-C1. The phenols have a simple benzene ring with an -OH group attached. Phenolic acids, such as hydroxybenzoic acids, are a subclass of phenolic compounds and their structures have at least one carboxylic acid group in addition to hydroxyl groups on the aromatic ring(Fig. 1). In addition to hydroxybenzoic acid derivatives, hydroxycinnamic acid derivatives (C6-C3), known as phenyl propanoids, are often categorized as phenolic acids. The latter is characterized by a 2-propenoic acid (acrylic acid) group attached to the phenol ring (Cianciosi et al., 2022; da Silva et al., 2023). These phenolic acids are generally found in a variety of fruits, vegetables, legumes, cereals, and their byproducts (Table 2); they mainly participate in the defence mechanism and protect plants from pathogens, microbes, and other environmental factors.

**Table 2:** Examples of phenolic acids in different plant species

Phenolic acids	Example	Common plant species	References
Hydroxybenzoic acid	*p*-hydroxybenzoic acid	• *Daucus carota* • *Elaeis guineensis* • *Vitis vinifera* • *Fagara macrophylla* • *Xanthophyllum rubescens* • *Vitex negundo* • *Areca catechu* • *Roystonea regia* • *Mespilus germanica* • *Hylocereus undatus (syn. Selenicereus undatus (Haw.) D.R.Hunt* • *Phaseolus vulgaris* L • *Glycine max* (L.)* Merr* • *Allium cepa* L	Chaudhary et al., 2013; da Silva et al., 2023; Li et al., 2019a, 2019b; Ma et al., 2022
	Gallic acid	• *Vitis vinifera* L • *Anacardium occidentale* L • *Corylus avellana* L • *Fragaria 9 ananassa (syn. Fragaria ananassa (Duchesne ex Weston) Duchesne ex Rozier)* • *Allanblackia floribunda* • *Syzygium cordatum* • *Tamarix nilotica* • *Toona sinensis* • *Hamamelis virginiana* • *Green tea*	da Silva et al., 2023; Özcan et al., 2018; Ragusa et al., 2019; Uslu & Özcan, 2019; Wianowska & Olszowy-Tomczyk, 2023; Zhou et al., 2022
	Vanillic acid	• *Musa acuminata* • *Mangifera indica* • *Euterpe oleracea* • *Angelica sinensis* • *Solanum tuberosum* • *Gossypium mexicanum* • *Melia azedarach* • *Panax ginseng* • *Lentinula edodes*	Kim et al., 2019a, 2019b; Kiokias et al., 2020; Siriamornpun & Kaewseejan, 2017; Zhao et al., 2018
	Syringic acid	• *Phoenix dactylifera* L • *Euterpe oleraceae Mart* • *Apis* • *Hordeum vulgare* • *Glycine max* • *Citrus spp.* • *Thymus vulgaris* • *Rosmarinus officinalis* • *Hibiscus tiliaceus* • *Eleusine coracana* • *Syzygium aromaticum*	Al Harthi et al., 2015; da Silveira & Godoy, 2019; Imtara et al., 2019; Liu et al., 2018a, 2018b, 2018c
	Protocatechuic acid	•*.Pisum sativum* • *Allium cepa* L • *Ribes uva-crispa* L • *Theobroma cacao* L • *Ginkgo biloba* L • Grape wine • Barley tea • Green tea	Nambiar et al., 2016; Oracz et al., 2019; Orsavová et al., 2019; Ražná et al., 2020; Sarikurkcu et al., 2020; Stodolak et al., 2020; Tao et al., 2019; Zhang et al., 2021
	Ellagic acid	• *Fragaria* × *ananassa (Weston) Duchesne ex Rozier* • *Juglans nigra* L • *Psidium guajava* L • *Rubus idaeus* L • *Rubus ursinus* × *Rubus idaeus* • *Vaccinium spp.* • *Vitis rotundifolia Michx*	Evtyugin et al., 2020; dos Santos et al., 2017; Williams et al., 2014
	Gentisic acid	• *Amaranthus caudatus* • *Citrus spp.* • *Vitis vinifera* • *Sesamum indicum* • *Gentiana spp.* • *Pterocarpus santalinus* • *Eucalyptus grandis* • *Saxifraga spp.* • *Olea europaea*	Li et al., 2015; Sarker & Oba, 2019
Hydroxycinnamic acid	*p*-Coumaric acid	• *Fragaria* × *ananassa* • *Pyrus communis* • *Musa paradisiaca Linn* • *Mangifera indica* • *Arachis hypogaea* • *Allium cepa* • *Agaricus bisporus* • *Amaranthus cruentus* • *Honey*	Bento-Silva et al., 2020; Nešović et al., 2020; Paśko et al., 2008; Pei et al., 2016; Siriamornpun & Kaewseejan, 2017
	Caffeic acid	• *Mangifera indica* • *Eucalyptus globulus* • *Ilex paraguariensis* • *Vaccinium sect. Cyanococcus* • *Salvia Rosmarinus* • *Ocimum basilicum* • *Salvia officinalis* • Coffee	Deotale et al., 2019; Dezsi et al., 2015; Kiokias et al., 2020; Mancuso & Santangelo, 2014; Meinhart et al., 2019
	Ferulic acid	• *Linum usitatissimum* • *Cynara cardunculus* • *Solanum melongena* • *Beta vulgaris* L • *Spinacia oleracea* • *Arachis hypogaea* • *Mangifera indica* • *Phaseolus vulgaris* • *Acai (Euterpe oleracea) oil* • Cell walls of cereal grains • Bamboo shoots	Kiokias et al., 2020; Kumar & Pruthi, 2014; Mancuso & Santangelo, 2014; Sgarbossa et al., 2015; Siriamornpun & Kaewseejan, 2017
	Sinapic acid	• *Brassica nigra* • *Brassica oleracea var. italica ‘Cezar’)*	Moreno-González et al., 2021; Olszewska et al., 2020
	Chlorogenic acid	• *Prunus avium* • *Malus domestica* • *Pyrus communis* • *Solanum melongena* • *Daucus carota* • *Brassica oleracea* • *Capsicum annuum*	Alarcón-Flores et al., 2014; Hallmann & Rembiałkowska, 2012; Lu et al., 2020; Mennella et al., 2012

The carbon skeleton of lignans is (C6—C3)_2_ and composed of two units of phenylpropane linked by β-β′ bond that is formed between the two carbon atoms of the side chains. They are often referred to as phytoestrogens due to the structural similarity to estrogen, which significantly influences the biological activity (Fig. 1). Lignans are classified into eight subgroups based on oxygen incorporation and cyclization patterns. These subgroups include aryl naphthalene, aryl tetralin, furan, furofuran, dibenzyl butane, dibenzyl butyrolactone, dibenzo cyclooctadiene, and tetrahydrofuran (Cianciosi et al., 2022; Rodríguez-García et al., 2019). Lignans are present in whole grains, oilseeds, legumes, fruits, and vegetables, among which sesame seeds are the richest source (Table 3). Lignans act as a plant protectant against microbes, fungi, pests, and insects (Ražná et al., 2021).

**Table 3:** Examples of lignans present in different plant species

Lignans in the food matrix	Common plant species	References
Matairesinol	• *Pinus pinaster* • *Vitis euvitis vinifera* L • Brazilian brown propolis	Dadáková et al., 2021; Gabaston et al., 2017; Ribeiro et al., 2021
Sesamin	• *Sesamum indicum* • *Zanthoxylum tetraspermum* • *Cuscuta palaestina* • *Acanthopanax sessiliflorus*	Abu-Lafi et al., 2018; Haque et al., 2016; Hata et al., 2013; Pullela et al., 2006; Sohel et al., 2022
Secoisolariciresinol	• *Actinidia chinensis Planch* • *Pinus pinaster* • *Linum usitatissimum* L • Soya oil	Khalid et al., 2022; Zhang et al., 2022
Lariciresinol	• *Vitis euvitis vinifera* L • *Citrus paradisi Macfad* • *Prunus avium* L • *Citrus reticulata Blanco* • *Vitis vinifera* L • *Solanum lycopersicum* • *Sonneratia apetala* • *Turrea nilotica* • *Monechma ciliatum* • Chocolate	Li et al., 2023
Pinoresinol	• *Pinus pinaster* • *Sesamum indicum* • *Citrus paradisi Macfad* • *Solanum lycopersicum* • Chocolate • Tofu • Olive oil	Gabaston et al., 2017; Pal et al., 2020

Stilbenes have the carbon skeleton C6-C2-C6, and their chemical structure consists of two benzene rings connected by an ethylene group (Fig. 1). Grapes are the major source of stilbenes, mainly resveratrol. However, resveratrol has been identified in at least 70 species. Plants produce stilbenes when injured or attacked by microbes, especially fungi, to protect themselves (Cianciosi et al., 2022; Valletta et al., 2021). Three predominantly found stilbenes in food are resveratrol, pinosylvin, and piceid (Table 4).

**Table 4:** Examples of stilbenes present in different plant species

Stilbene derivatives in the food matrix	Common plant species	References
Resveratrol	• *Vitis vinifera* L • *Morus alba* L • *Veratrum maackii Regel* • *Rumex japonicus Houtt* • *Ananas Tourm. ex Linn* • *Theobroma cacao* • *Arachis hypogea* • *Artocarpus heterophyllus*	Magrone et al., 2017; Riccio et al., 2020; Tian & Liu, 2020
Pinosylvin	• *Pinus sylvestris* • *Hovenia dulcis Thunb* • *Picea glauca* • *Nothofagus (Southern beeches)* • *Stemona cf. pierrei* • *Arachis hypogaea*	Bakrim et al., 2022; Gabaston et al., 2020; Gyeltshen et al., 2022; Koo et al., 2022; Lim et al., 2015; Vek et al., 2020; Verkasalo et al., 2021
Piceid	• *Vitis vinifera* • *Theobroma cacao* L	Cheynier et al., 2010; Basholli-Salihu et al., 2016

## Carbohydrates: an overview

### Carbohydrates within the plant cells

**Fig. 2:**
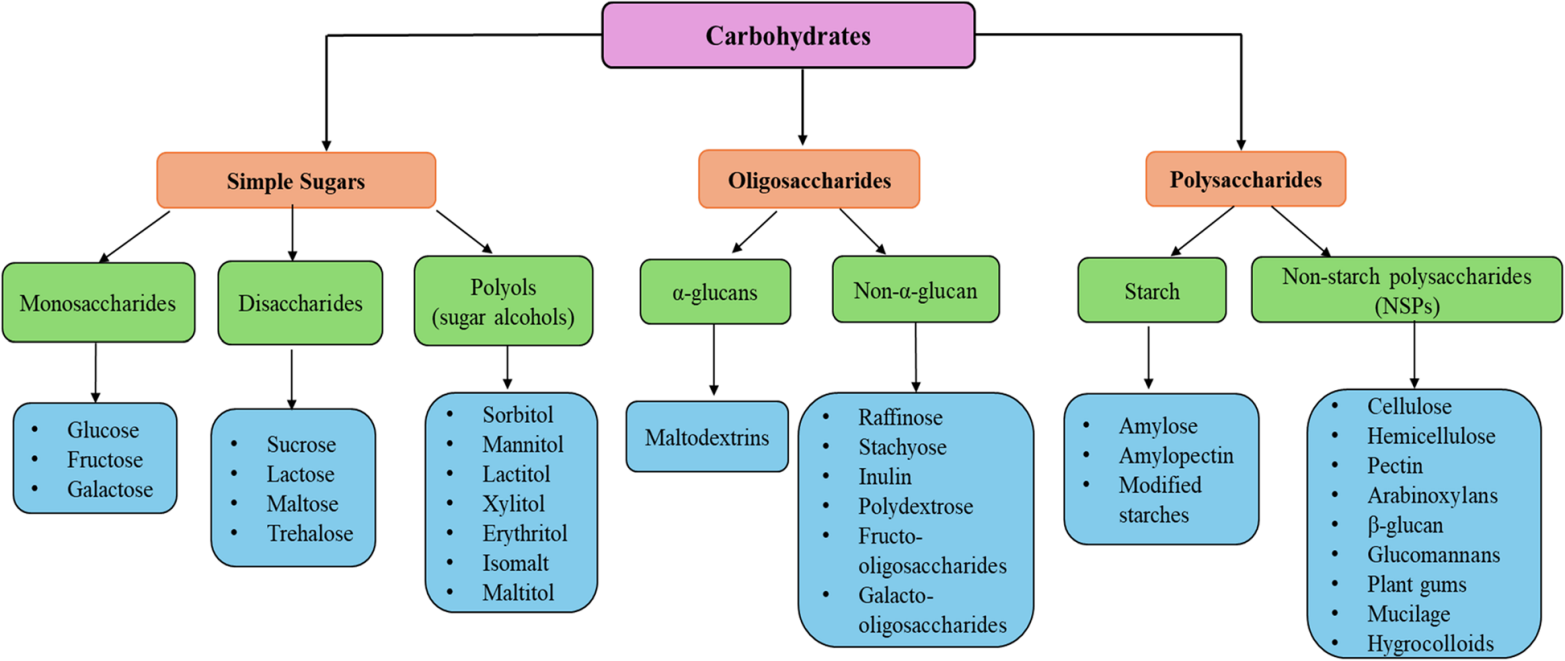
Overview of carbohydrate classes: simple sugars, oligosaccharides, and polysaccharides (Created using Microsoft PowerPoint)

Carbohydrates have the general formula of Cx(H_2_O)y. In addition to being an essential source of energy for living organisms, they also function as a reliable means of energy storage (Großkopf & Simm, 2020). Carbohydrates are divided into major subgroups, including monosaccharides, disaccharides, oligosaccharides, and polysaccharides (Fig. 2).

The classification is based on factors such as molecular size, degree of polymerization (DP), type of linkage, and the characteristics of the individual monomers. Monosaccharides are monomeric carbohydrate molecules with a DP of 1, such as glucose and fructose. Disaccharides are made of two monosaccharides linked by a glycosidic bond and have a DP of 2, such as lactose and sucrose. At the same time, oligosaccharides are short-chain carbohydrates formed by three or more monosaccharides connected by glycosidic linkages and have a DP ranging between 3 and 9, such as raffinose and stachyose. Meanwhile, polysaccharides are regarded as long-chain carbohydrates, formed by ten or more monosaccharide units, DP ≥ 10, such as starch and cellulose (Dilworth et al., 2024).

Dietary carbohydrates are ingested from food and influence human health in many ways. They affect blood glucose and insulin levels, lipid metabolism, and satiety, and influence bowel and colon functions by being fermented by gut microbiota (Cummings & Stephen 2007). Carbohydrates, especially starch, are found in most cereals, such as rice, wheat, and barley (Table 5). They are also found in legumes, fruits, and vegetables such as bananas and potatoes (Tables 6 and 7).

**Table 5:** Carbohydrates (starch, total simple sugars, and fiber) (in %) in various cereal grains

Cereals	Starch	Total simple sugars	Fiber	References
*Oryza sativa*	72.94–73.45	6.10–6.15	3.9–4.2	Das et al., 2012; Devindra et al., 2011
*Triticum aestivum*	68.1	3.1	12	Das et al., 2012; Escarnot et al., 2012
*Zea mays*	70–75	4.5–5.6	15	Das et al., 2012; Szymanek et al., 2015
*Sorghum bicolor*	52.70	9.80–11.11	10.7	Das et al., 2012; Permanasari et al., 2017; Rono et al., 2018
*Hordeum vulgare*	50–56	3.3–3.5	10	Das et al., 2012

Total simple sugars include, butnot limited to, glucose, fructose, and sucrose; mannose and galactose may be present in trace amounts, depending on the species

**Table 6:** Carbohydrates (starch, total simple sugars, and fiber) (in %) in different legume species (Adapted from Hedley 2001)

Legume species	Starch	Total simple sugars	Fiber
*Glycine max L. Merr*	1.5	32.5	20
*Cicer arietinum L*	44.4	65.3	9
*Lupin spp.*	0.4	36.7	26
*Vigna radiata*	45.0	60.0	7
*Cajanus cajan Millsp.*	44.3	64.9	10
*Phaseolus vulgaris L*	41.5	61.3	10
*Canavalia ensiformis*	35.0	47.8	9
*Vicia faba*	41.0	59.8	12
*Pisum sativum*	45.0	65.5	12
*Lens culinaris*	46.0	64.4	12

Total simple sugars include, but not limited to, glucose, fructose, sucrose, galactose, and mannose. Trace amounts of oligosaccharides such as raffinose and stachyose may also be present, depending on the species

**Table 7:** Carbohydrates (starch, total simple sugars, and fiber) (in % DW) in different fruits and vegetables

Plant species	Starch	Total simple sugars	Fiber	References
**Vegetables**				
* Solanum tuberosum*	61–80	4.8	9.6 (TDF)	Chandrasekara & Josheph Kumar, 2016
* Manihot esculenta*	74–87	4.4–5.0	3.4 (C)	Lebot et al., 2019; Mejía‐Agüero et al. 2012
* Ipomea batatas*	65–70	10.2	2.6–4.2 (C) 0.5–4.5 (CF)	Neela & Fanta, 2019; Oliveira et al., 2019; Otegbayo et al., 2012; Wireko-Manu et al., 2011
* Dioscorea cayenensis-rotundata*	70–86	1.9–4.6	0.51 (CF) 2.1 (C)	Lebot et al., 2019; Njoh Ellong et al., 2015; Otegbayo et al., 2012; Vanessa Ezeocha et al., 2012
* Dioscorea dumetorum*	68	1.2–3.8	2.9–3.7 (C)	Rinaldo, 2020
* Dioscorea alata*	65–76	2.1–5.7	2.3 (CF) 14 (TDF)	Champagne et al., 2009
* Colocasia esculenta*	78	5.0–11.3	2.1 (C)	Champagne et al., 2009
* Xanthosoma sagitifolium*	86	2.2	3.3 (C)	Rinaldo, 2020
**Fruits**				
* Musa sp*	44–61 (MG) 2–6 (R)	0.5 (MG) 12–16 (R)	2.1–4.4 (TDF)	Soares et al., 2011
* Musa paradisiaca*	74–80 (MG) 28–41 (R)	0.7–1.6 (MG) 9.0 (R)	2.8 (TDF) 1.4–1.8 (CF)	Fils-Lycaon et al.,^ 2011^; Khawas et al., 2014; Soares et al., 2011
* Artocarpus altilis*	77 (MG)	-	-	Bramont et al., 2018; Clerici & Carvalho-Silva, 2011; Englberger et al., 2014
* Artocarpus heterophyllus*	79 (MG)	-	8.9 (CF, MG) 7.3 (CF, R)	Bramont et al., 2018; JurezBarrientos et al., 2017; Madrigal‐Aldana et al. 2011

*Abbreviations*: *MG* Mature green, *R* Ripe, *TDF* Total dietary fiber, *CF* Crude fiber, *C* Cellulose

Total simple sugars include, but are not limited to, glucose, fructose, and sucrose. Maltose and galactose may also be present in trace amounts, depending on the species

### Carbohydrates in plant cell walls

The primary and secondary cell walls, gelatinous layer walls (in some species), serve as the physical barrier between the components of the cell and the environment (Liu et al., 2020). The plant cell wall is the primary determinant of the texture of plant-based food, and it also serves as a significant source of dietary fiber.

**Fig. 3:**
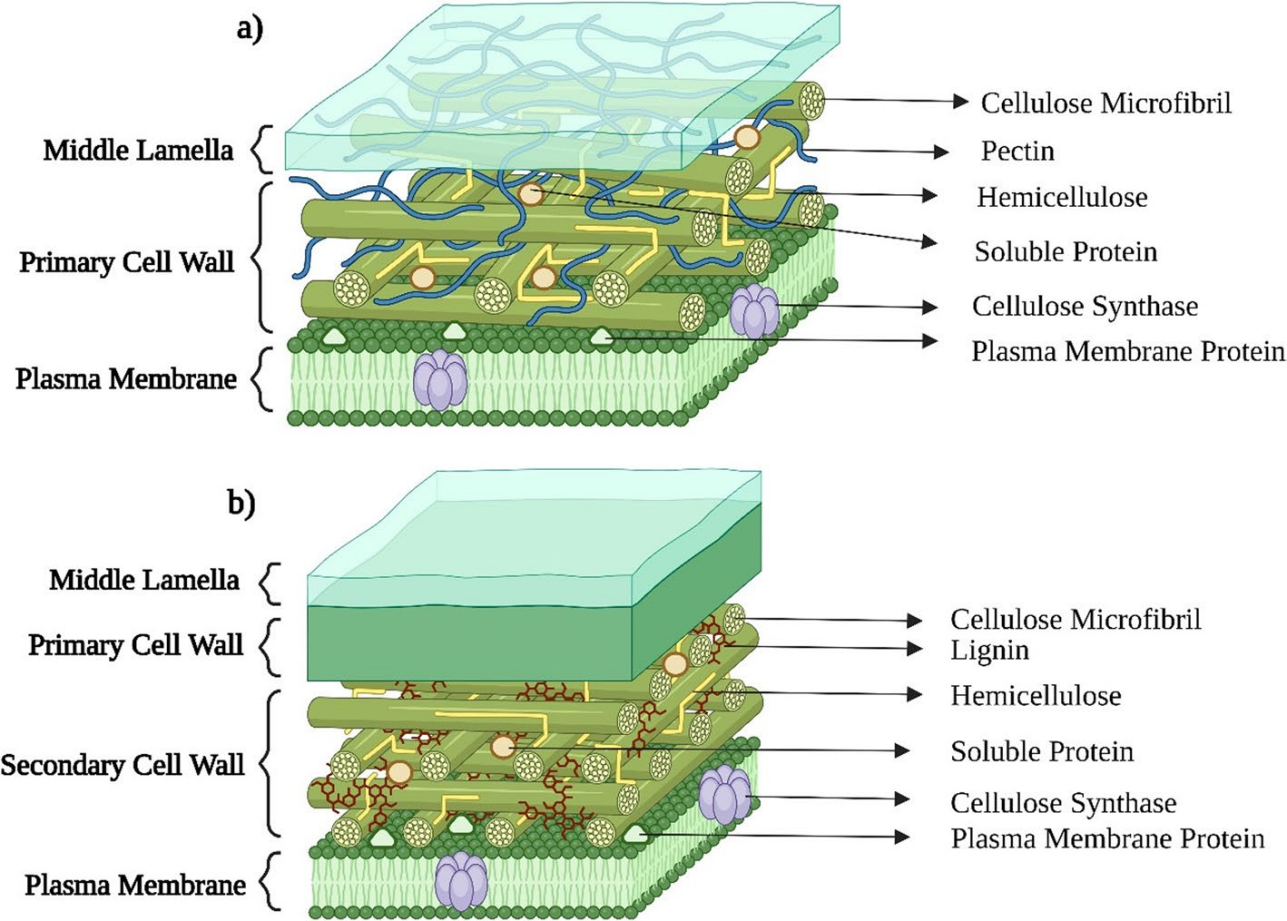
Structural composition of plant cell walls: Primary cell wall (**a**) and Secondary cell wall (**b**) (Created using Biorender)

The primary cell wall comprises carbohydrates such as hemicellulose, cellulose, and pectin. The secondary cell walls are formed with phenolic components such as lignin via ester and ether bonds within the organized polysaccharide structure (Fig. 3) (Ebringerová, 2006; Krzesłowska, 2011). The hemicellulose is attached to the cellulose microfibril through hydrogen bonds, and it may further be divided into four subgroups, namely xylans, xyloglucans, glucomannans, and mixed-linkage glucans. Pectins are also attached to the cellulose-hemicellulose network by forming a hydrated gel structure, and they have four major domains such as xylogalacturonan (XGA), rhamnogalacturonan I (RGI), rhamnogalacturonan II (RGII), and homogalacturonan (HGA). The secondary cell wall has a similar composition except for lignin. During secondary wall formation, the relative content of pectin decreases while lignin is deposited, resulting in a more rigid and hydrophobic structure. Lignin consists of three subunits, namely G (guaiacyl)-, S (syringyl)-, and H (p-hydroxyphenyl)-lignin (Liu et al., 2020; Loix et al., 2017).

During food processing, the carbohydrates in the cell wall, especially pectins, are more susceptible to physical and chemical conversion reactions, which ultimately affect the structure and even alter their functional properties (Christiaens et al., 2016). The alterations during food processing include changes in porosity and specific areas within the cell wall (Le Bourvellec et al., 2005; Liu et al., 2020). The presence of polyphenols within the cell wall also involves altering the functional properties, including changes in the food systems'rheological properties, texture, and even stability (Dobson et al., 2019; Tudorache & Bordenave, 2019).

The formation of polysaccharide-polyphenol aggregates by polyphenols can cause a decrease in polysaccharide viscosity and pseudo-plasticity (Tudorache et al., 2020). A better understanding of polysaccharide-polyphenol interaction could lead to the development and improvement of polysaccharide-polyphenol complex-specific applications in the food industry.

## Interaction between polyphenols and polysaccharides

**Fig. 4:**
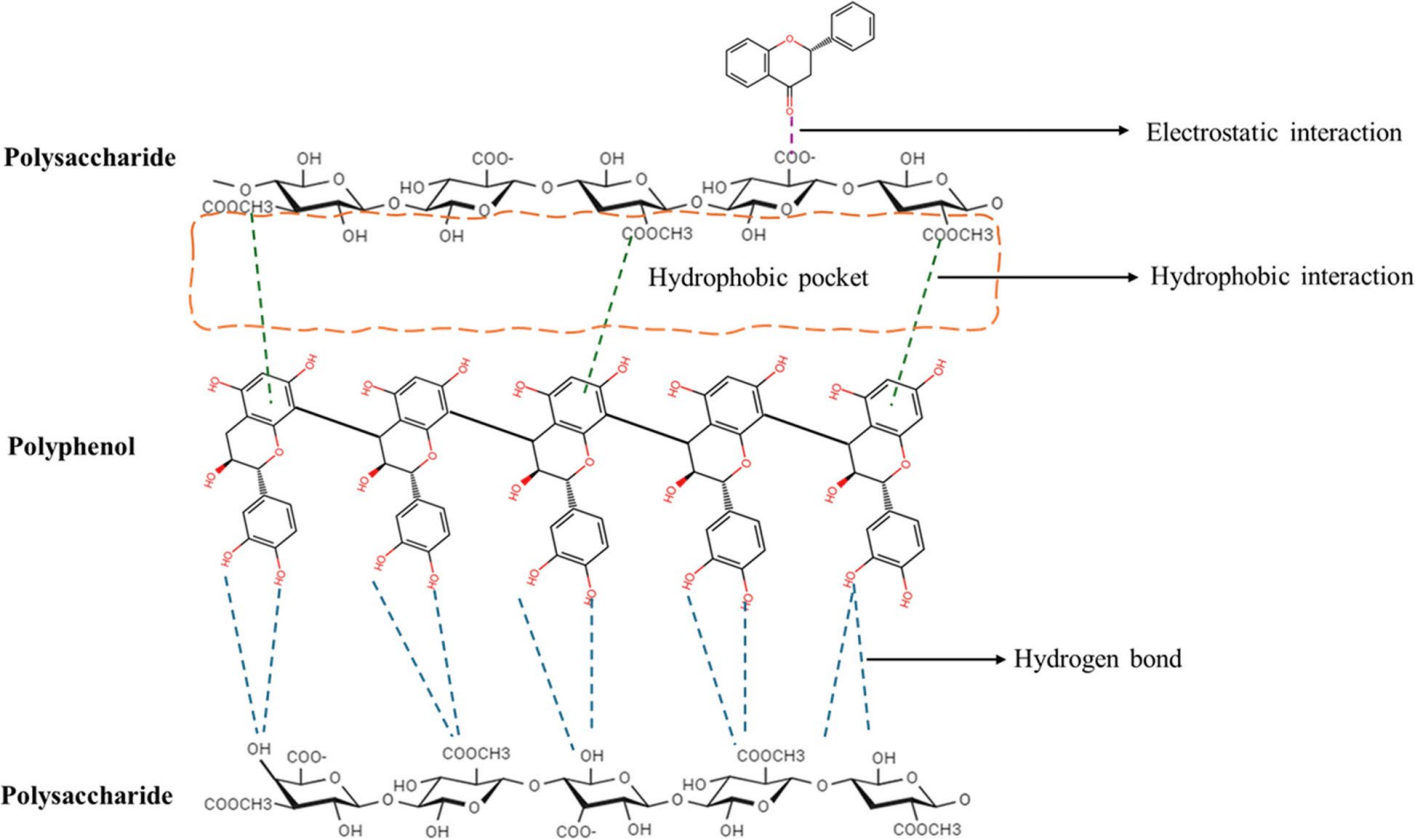
Different interactions (electrostatic, hydrophobic and hydrogen bonding (carbonyl-hydroxyl or hydroxyl-hydroxyl) interactions) between polysaccharides and polyphenols (Created using ChemSketch and Microsoft PowerPoint)

The food matrix comprises various biomolecules, including proteins, carbohydrates, lipids, and polyphenols. These biomolecules often interact with each other, which alters their bioactivity, bioavailability, and biological efficacy (Holland et al., 2020; Ribas-Agustí et al., 2018). Polyphenols interact with polysaccharides through both covalent and non-covalent interactions. The non-covalent interactions or conjugation involve the formation of weak bonds such as hydrogen bonds, van der Waals forces, and hydrophobic bonds between both biomolecules (Fig. 4). For example, galacturonic acid's methoxy group (a prevalent hydrophobic site) engages with polyphenols'aromatic rings through hydrophobic interactions (Liu et al., 2020).

External factors such as temperature extremes, pH variation, and chemical agents like urea significantly affect polyphenol-polysaccharide interactions. These factors predominantly influence non-covalent interactions, such as hydrogen bonding and hydrophobic interactions. Urea, for example, interferes with hydrogen bonding by affecting the polyphenol's hydroxyl groups and oxygen atoms of polysaccharide glycosidic linkages, ultimately weakening the complex. Covalent bonds are formed under such stress conditions, where non-covalent bonding is disrupted, especially under oxidative conditions. In these cases, oxidized polyphenols (such as quinones) can form covalent linkages with nucleophilic groups on polysaccharides. This shift from non-covalent to irreversible covalent interactions alters the stability and functional properties of the complexes (Le Bourvellec et al., 2005; Renard et al., 2001).

Interestingly, some polysaccharides, such as cyclodextrins (CD), naturally exist as cylindrical structures. The CDs are cyclic oligosaccharides with a hydrophobic cavity and hydrophilic outer surface derived from amylose and exist in several forms: α-cyclodextrin, β-cyclodextrin, and γ-cyclodextrin with six, seven, and eight glucose units, respectively, linked by α-(1–4) glucosidic linkage (Connors, 1997; Le Bourvellec & Renard, 2012). Most polyphenols typically have low water solubility and tend to form inclusion complexes with polysaccharides such as CDs, which possess a hydrophobic central cavity. These interactions improve polyphenols'solubility, stability, and bioavailability through non-covalent hydrophobic interactions (Liu et al., 2020).

Researchers have studied the interaction between polyphenols and polysaccharides and their effects, which are listed in Table 8. Though these interactions have been extensively investigated, their mechanism of complex formation and what happens afterward must be elaborated more precisely, as some studies show contradictory results.

**Table 8:** Summary of interactions between different polysaccharides and polyphenols

Polysaccharide	Dataset	Polyphenol	Interaction	Observation	References
Pectins from citrus and apple	30% and 70% degree of methylation (DM) of homogalacturonan (HG)	Procyanidins of apple with the degree of polymerization (DP) of 9 and 30	Hydrophobic interaction and hydrogen bonds	HG with high DM has a strong hydrophobic interaction with the polysaccharide (procyanidins)	Watrelot et al., 2013
Pectins from citrus	Different types and degrees of esterification (DE): AHG 30%, HG 30%, HG 70%, HG-B 70%, and HG-R 70%	Cyanidin-3-*O*-glucoside	Hydrogen bond, electrostatic and hydrophobic interaction, and π-π stacking	Cyanidin-3-*O*-glucoside binds with the polyphenol in the order: HG 30% > AHG 30% > HG-B 70% ~ HG-R 70%	Fernandes et al., 2020
Pectins from citrus peel	High and low methoxy pectin, DE74 and DE30, respectively	Tannins with DP 5 and 26	Hydrophobic interaction and hydrogen bond	DE74 > DE30	Mamet et al., 2018
Pectins from citrus, apple, and sugar beet	LM, HM, and AM	Anthocyanins and non-anthocyanin phenolics from black currant	Electrostatic and hydrophobic interaction, hydrogen bond	• AM > LM > HM • The source of pectin plays an important role in interaction, which follows the following order: citrus > apple > sugar beet	Buchweitz et al., 2013a
Pectins from citrus, apple, and sugar beet	LM, HM, and AM	Anthocyanins from strawberry	Hydrogen bond	• The degree of amidation (DA) and degree of esterification (DE) of pectins does not affect the stability of anthocyanins • The citrus pectin does not enhance the stability of anthocyanins • Pectins from apple and sugar beet enhance the stability of anthocyanins	Buchweitz et al., 2013b
Pectins with different neutral sugar side chains	Hairy fraction of pectins	Procyanidins from Apple with DP9 and DP30	Hydrophobic interaction and hydrogen bonds	The interaction between procyanidins and pectins is limited due to the presence of complex neutral sugar side chain components	Watrelot et al., 2014
Arabinan-rich pectic polysaccharides (ARPPs)	Arabinans from apple and sugar beet	Procyanidins, phloridzin, and chlorogenic acid	Hydrophobic interaction and hydrogen bond	Interaction between the polyphenols and arabinan was limited due to the presence of a higher degree of branching of arabinan	Fernandes et al., 2020
ARPPs	Different molecular weights	27 phenolic acid monomers	Non-covalent interaction	The affinity of ARPP increases in the order: P4 (153 kDa) < P3 (24 kDa) < P1 (114 kDa) < P2 (76 kDa)	Zhu et al., 2018
Pectins from blueberry	Chelator-soluble pectin fraction (CSF) and water-soluble pectin fraction (WSF)	Anthocyanins	Electrostatic and hydrophobic interactions, hydrogen bonds, and π-π stacking	Stronger interaction between the anthocyanins and pectins when the pectins are highly linear, neutral-sugar-rich (WSF) and negatively charged pectin (CSF) than more-branched,	Koh et al., 2020
Pectins from blueberry	WSF, CSF, and carbonate-soluble fractions (NSF)	Anthocyanins	Electrostatic interaction and intermolecular stacking	• Interaction is pH dependent • Strong interaction between anthocyanins of- • NSF when pH is between 3.6 and 4.5 • CSF when pH is between 2.0–3.6 • WSF has poor binding ability	Lin et al., 2016
Pectin rich in iron	HM pectin with Fe^2+^ and Fe^3+^	Rutin and quercetin	Interaction occurs via iron ions as a mediator	Iron-rich pectins have a higher affinity for rutin and quercetin	Chirug et al., 2018
Pectin from sugar beet	Pectins modified by ultrasound treatment or an enzyme	Anthocyanins	Hydrophobic interaction, hydrogen bonds, and van der Waals interaction	• Insoluble anthocyanin-pectin complexes are formed with high molecular weight pectins • Soluble complexes are formed using ultrasound or enzyme-modified pectin	Larsen et al., 2019
The cell wall of strawberry	Drying kinetics was studied with the cell wall analyzed after oven (60 ºC)and freeze-drying (80 ºC)	3,4- dihydroxyphenylglycol (DHPG) and hydroxytyrosol (HT) from olive	Electrostatic interaction, hydrogen, and ester bond	The interaction was enhanced by drying the strawberry cell wall	Bermúdez-Oria et al., 2019
The cell wall of a pear	Overripe, ripe, whole flesh (WF), parenchyma cells (PC), skin (SK), and stone cells (SC)	Procyanidin in pear	Hydrophobic interaction and hydrogen bond	• Overripening of pears enhances the adsorption of procyanidins on the cell wall of pears (maybe due to the absence of pectic side chains) • The bound procyanidins proportion increases in the following order: PC > WF > SC > SK	Brahem et al., 2019
The cell wall of grapes	Ripe and veraison grape cell walls	Proanthocyanidins (DP3) from grape skin	Non-covalent interaction	The ripe grape cell walls retain more proanthocyanidins than the grape cell walls	Castro-López et al., 2016
The cell wall of grapes	Insoluble and soluble cell wall fractions	Proanthocyanidins (DP21) from grape skin	Non-covalent interaction	• The treatment using polygalacturonase reduced proanthocyanidin adsorption by the mesocarp cell walls • Compared with native skin cells, the pectic fraction reduces the adsorption of procyanidin	Bindon et al., 2016
The cell wall of grapes	Comparison between different extraction solvents such as CDTA, 50 mM, 1 M, and 4 M KOH	Proanthocyanidins from the skin of grapes	Non-covalent interaction	The CDTA solvent had the most cell wall-bound proanthocyanidin	Ruiz-Garcia et al., 2014
Grape skin and flesh cell walls	Different grape maturity stages	Proanthocyanidins from the skin of grapes	Non-covalent interaction	• Flesh skin walls interact and bind more proanthocyanidin than that from skin • Adsorption capacity of the grape skin cell wall decreases remarkably upon maturation • Adsorption capacity of the flesh cell wall did not affect much upon maturation	Bindon et al., 2012
The cell wall of grapes	Skin and flesh of grapes	Proanthocyanidins from the skin of different stages of ripened grapes	Non-covalent interaction	The flesh cell wall interaction was higher than that of the skin cell wall of grapes	Bindon & Kennedy, 2011
The cell wall of grapes	Skin and flesh of grapes	Proanthocyanidins from the skin and seed of grapes	Non-covalent interaction	The flesh cell wall interaction was higher than that of the grape's skin cell wall	Bindon et al., 2010
The cell wall of an apple	Starch, cellulose, pectin, and xyloglucan	Procyanidins derived from pear, apple, and grape seed	Non-covalent interaction	The intensity of the interaction follows the order: Pectin > > xyloglucan > starch > cellulose	Le Bourvellec 2005
The cell wall of an apple	Three varieties of apples, such as “Pink Lady”, “Granny Smith”, and “Red Delicious”, were obtained and followed by boiling, oven, and drying	Phloridzin, Epicatechin, and Chlorogenic acid	Hydrophobic interaction and hydrogen bond	• Among the different varieties of apple, “Pink Lady” expressed the highest binding affinity towards the polyphenols due to the presence of high levels of uronic acid in its pectin • The binding order of the polysaccharide towards the polyphenol is: Fresh≈boiled > oven-dried > freeze-dried	Liu et al., 2019a, 2019b, 2019c
The cell wall of an apple	Native and modified cell wall (modification is done via mild or harsh drying)	Procyanidins from apple. Pear and grape seed	Hydrophobic interaction and hydrogen bond	The affinity of the cell wall towards procyanidins decreases upon harsh drying	Le Bourvellec et al., 2005
The cell wall of an apple	The cell wall was modified using drying and boiling	Procyanidins	Hydrophobic interaction and hydrogen bond	• Both boiling and drying decreased the affinity of the interaction but increased the saturation level in the cell wall • Cell wall protein doesn’t contribute to the interaction between the cell wall polysaccharide and procyanidin	Le Bourvellec al. 2012
Wheatstarch	Different mass ratios and concentrations of wheat starch to wheat starch	Tannic acid	Non-covalent interaction	The formation of tannic acid/wheat starch complexes increases with an increase in tannic acid/wheat starch mass ratio	Wei et al., 2019
Maize starch	Different ratios of amylose/amylopectin	Oligomeric procyanidins	Hydrogen bonds	High amylose maize starch exhibits stronger interaction with oligomeric procyanidins than with that of high amylopectin or normal maize starch	Liu et al., 2017a, 2017b
Starch	High amylose, waxy and normal starches, pure amylose and amylopectin,	Phenolic compounds of sorghum and tannins	Chemical and hydrophobic interaction, hydrogen bonds	Interaction between pure amylose with oligomeric and polymeric proanthocyanidins	Barros et al., 2012
Xylan and cellulose	Dietary fibers	Caffeic acid, catechin, and ferulic acid	Non-covalent interaction	Both the polysaccharides exhibited low adsorption to all three polyphenols	Costa et al., 2015
Pectin and cellulose	Structure and composition of the polysaccharide	Catechin	Non-covalent interactions, specifically physisorption	Cellulose exhibits a higher interaction than pectin with catechin	Liu et al., 2019a, 2019b, 2019c
Methylcellulose	Different molar ratios	Tannic acid and Epigallocatechin-3-gallate	Hydrogen bonds and hydrophobic interactions	21 mol of epigallocatechin-3-gallate and 33 mol of tannic acid exhibit a strong interaction with 1 mol of methylcellulose	Patel et al., 2013
Konjac glucomannan	Molecular weight altered using sonication	Tannic acid	Hydrogen bond	With an increase in molecular weight of konjac glucomannan, the Tannic acid/konjac glucomannan complex sizes decrease	He et al., 2019

## Parameters in polysaccharides influencing polysaccharide-polyphenol interaction

The various physical characteristics of polysaccharides influence the affinity of polyphenols and the interaction between polysaccharides and polyphenols. An in-depth analysis of these factors is presented in the following sections.

### Surface area or porosity

**Fig. 5:**
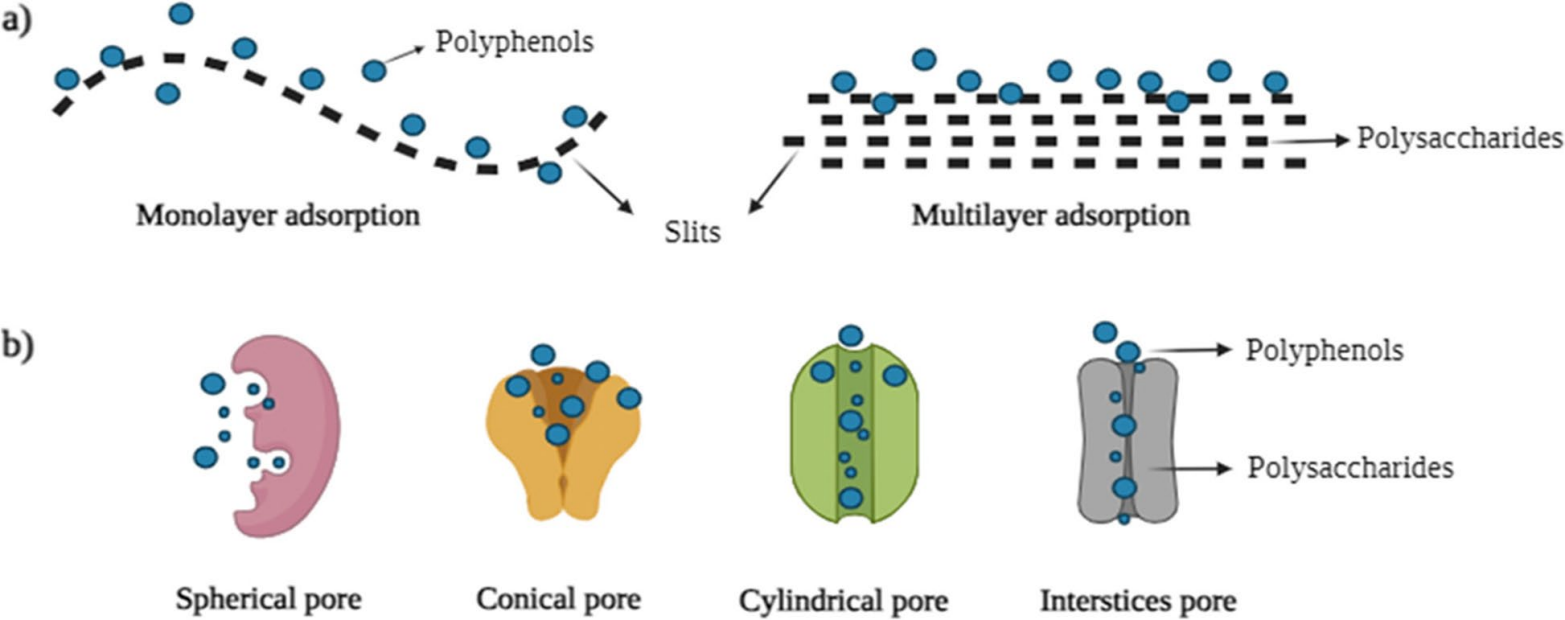
Different types of adsorption (**a**) and diffusion (**b**) within the cell wall matrix (created using Biorender)

Diffusion is considered one of the critical pathways for transporting polyphenol molecules into the polysaccharides during the interaction. There are many types of diffusion, primarily diffusion of molecules on the surface (surface adsorption or film diffusion) and pore diffusion inside the cell walls (Zhang et al., 2016) (Fig. 5). Generally, an optimal pore size of the cell walls would be approximately 5 nm, limiting the adsorption of polyphenols with molecular weight ≥10 kDa. To overcome this drawback, researchers have modified the cell wall characteristics, such as changes in porosity and surface area of polysaccharides, using physical methods and improving the adsorption efficiency of polyphenols. Two intrinsic factors are positively correlated: porosity and surface area. The larger the surface area, the more porous the cell wall (Bermúdez-Oria et al., 2019; Liu et al., 2017a, 2017b, 2019a, 2019b, 2019c).

Le Bourvellec et al. (2005) conducted an experiment using two different drying techniques (mild and harsh) that caused differences in the adsorption capacity of the cell wall material (similar composition of the cell wall material without drying was compared). Severe/harsh drying reduced the surface area of the cell wall material, which collapsed the network and reduced the adsorption of polyphenol (procyanidin). As a result, fewer pores were available in the cell wall material for the adsorption of polyphenols, and reduction or loss of porosity correlated with low or no binding sites for the polyphenols in the cell wall.

Liu et al., (2017a, 2017b) employed freeze-drying as a drying technique, significantly reducing the surface adsorption of polyphenols onto cellulose. This effect may be attributed to removing water molecules during freeze-drying, which decreases the surface area and subsequently lowers polyphenol adsorption. Certain fruits, such as grapes, undergo cell wall remodeling during ripening. In grapes, ripening increases cell wall porosity, leading to enhanced adsorption of proanthocyanidins (Bindon et al., 2012); fruits such as pears possess stone cells in their secondary cell wall which is less porous, flexible, and have less affinity towards procyanidins compared to the primary cell walls which adsorb higher procyanidins (free energy of adsorption in the range of ΔG = −15.90 to −22.98 kJ/mol) due to their flexible and porous cell wall material (Brahem et al., 2019). It can be observed that the adsorption of polyphenols increases with increasing porosity and optimal alignment between pore size and adsorbed polyphenol in the insoluble cell wall materials.

### Molecular weight

The molecular weight of polysaccharides significantly impacts the interaction of polyphenols and polysaccharides, as reported by several researchers (Guo et al., 2018; He et al., 2019; Larsen et al., 2019; Zhu et al., 2018). The influence of molecular weight on the interaction of polysaccharides and polyphenols is debatable. Some studies have noted that high molecular weight polysaccharides interact and bind more polyphenol molecules. This observation was supported by a claim that these polysaccharides contain more glycosidic linkages and branched chains in their structures, whereby the possible binding sites for polyphenols are higher in number, which facilitates the interaction (Guo et al., 2018; He et al., 2019).

However, Zhu et al. (2018) obtained a contrasting result that showed no relationship between molecular weight and the affinity/binding of polyphenols. In their study, rapeseed meal polysaccharides of different molecular weights were analyzed. However, the composition of the polysaccharides was not considered, which suggests that the results drawn were inconclusive. The composition of polysaccharides has been considered an essential factor because each polysaccharide has its own structural conformation, whereby the affinity and adsorption of polyphenols differ. The adsorption of procyanidin on the pectin complex was reported to have high affinity due to a neutral side chain in the pectin structure. It was also noted that this side chain hinders the polyphenol adsorption on the surface by steric hindrance (Fernandes et al., 2020; Liu et al., 2020).

Further studies were conducted to analyze the importance of the presence of a side chain in polysaccharide structure during the interaction of polysaccharides and polyphenols. Larsen et al. (2019) used ultrasound and eliminated the rhamnogalacturonan I (RGI) side chain in pectins from sugar beet; this ultimately decreased the hydrodynamic volume. The decrease in the RGI side chain from the structure simultaneously increased the number of homogalacturonans (HGs) accessible in the pectin, which can interact with polyphenols (anthocyanins) and form a complex. Compared to the native pectins, the simplified framework facilitated a higher concentration of anthocyanins through self-copigmentation, which was achieved by having fewer branched RGs, which minimized the obstructive impact of side chains or entanglements on the binding of anthocyanins.

### Side chain and branching ratios

Several studies have explored the effects of the side chain, especially the neutral side chain of cell wall polysaccharides, and their influence on the adsorption of polyphenols. The branched structure of polysaccharides, due to the presence of neutral sugar side chains such as RGI and RGII, affects the binding and adsorption of polyphenols (Brahem et al., 2019; Koh et al., 2020). Watrelot et al. (2014) reported that the presence of neutral sugars such as RGI and RGII in the side chain of pectins limited the adsorption of procyanidin on the surface of pectin.

The degree of polymerization (DP) of procyanidin also played a pivotal role in their adsorption on the pectin surface. Procyanidin with DP9 had no significant difference in their interaction with pectin, but procyanidin with DP30 exhibited a clear difference; the difference in binding occurred in the following order: RG> arabinans+galactan I+xylogalacturonan > galactan I >arabinans+galactans II > arabinans. Pectins with linear structures and fewer neutral sugar side chains possessed greater adsorption capacity for procyanidins than pectins with higher branches. Another study by Koh et al. (2020) also concluded the same result with another polyphenol (anthocyanin). Anthocyanin was observed to associate and adsorb more to pectins with a more linear structure than branched ones.

Fernandes et al. (2020) studied the interaction of pectin and polyphenol. They concluded that the degree of arabinan side chain branching (high or low) in pectins affected the interaction. The globular structure of highly branched pectin side chains (arabinan) reduces the interaction of pectin with polyphenols. On the other hand, when the pectin molecule is non-branched (low arabinan), it is more linear and helical; this leads to the formation of hydrophobic domains by polymer entanglement and enhances the polyphenol encapsulation within the polysaccharide.

### Degree of esterification

The degree of esterification (DE) plays a significant role in the interaction of polysaccharides with polyphenols. Low or non-methylated pectin has a low affinity towards polyphenols (procyanidin) compared to that of highly methylated pectin, where the binding occurs via hydrophobic interaction with procyanidin (Watrelot et al., 2013). Several potential explanations exist for this phenomenon, including a higher hydrophobic interaction between the dihydropyran heterocycles present in procyanidins and methyl groups of pectins. After 24-hour exposure, pectins with high degrees of esterification (DE60) showed increased phenolic acid adsorption compared to low-DE pectin (DE30) (Padayachee et al., 2012a). Similarly, high methoxy pectins (DE74) exhibited a strong affinity towards tannins, which are highly polymerized (degree of polymerization-26) compared to the low-methoxy pectins (DE30) (Mamet et al., 2018).

Alternatively, the interaction between anthocyanins and pectins relies entirely on anthocyanin's positive charge. Buchweitz et al. (2013a) concluded that low-methylated and amidated pectins exhibited a stronger affinity towards the anthocyanins extracted from black currants than that of highly methylated pectins. An explanation could be the electrostatic interaction between the anthocyanin and the negatively charged carboxylate group in low-methylated pectins or with available electrons of the amide group in amidated pectins. Anthocyanin-3-*O*-glycoside exhibits stronger interaction with low-esterified pectins than with high-esterified pectins. The interaction between these polysaccharides and polyphenols is suggested to occur via hydrophobic interaction or hydrogen bonds (Fernandes et al., 2020).

Subsequently, Buchweitz et al. (2013b) found that the interaction between anthocyanins and pectins (obtained from different sources like apple, citrus, and sugar beet) differed based on the degree of amidation (DA) and DE. Apple and citrus pectins do not affect the stability of anthocyanins from strawberries. The stabilization of the anthocyanin was attributed to the hydrogen bond between the carboxyl and hydroxyl groups of pectin and the B-ring of anthocyanin (also depending on the number of hydroxyl groups in the B ring of anthocyanin).

### Presence of other molecules in the cell wall

The wall of plant cells contains many biomolecules other than polysaccharides (Fig. 3). The relation between these molecules and their impact on the interaction of polyphenols and polysaccharides has been widely analyzed. Water, a principal constituent of the cell wall, plays a pivotal role in the interaction, as was noted in the previous section on the importance of drying and adsorption of polyphenols. Similarly, an evaluation of the rehydration rate of freeze-dried samples of cellulose and the polyphenol adsorption capacity was also reported by Liu et al. (2017a, 2017b). One of the possible reasons could be the establishment of strong hydrogen bonds within the cellulose microfibres after drying, whereby the number of free hydroxyl sites for polyphenol binding in the cellulose is reduced.

Proteins present in the cell wall do not impact the adsorption of polyphenols on the polysaccharide surface; this was analyzed using procyanidins, where the same degree of interaction among the procyanidins and polysaccharides was found in the presence of low and high protein in the cell walls (Le Bourvellec & Renard, 2012). Studies have shown that higher lignin content in lignocellulosic material enhances the adsorption of catechins, which could be attributed to π–π stacking interactions and hydrogen bonding between the aromatic structures of lignin and the hydroxyl groups of catechins (Ye et al., 2009). Fernandes et al. (2020) demonstrated that apple arabinans, lacking ferulic acid, exhibit weaker interactions with procyanidins compared to sugar beet arabinans containing ferulic acid, highlighting the role of phenolic residues in facilitating polyphenol-polysaccharide interactions. However, in some cases, ferulic acid can also occupy potential binding sites or alter the spatial structure of the polysaccharide, which can inhibit further polyphenol adsorption. The effect of ferulic acid on binding is conditional and depends on factors such as concentration, structural characteristics of the polysaccharide, and the type of polyphenol involved in the interaction.

## Parameters in polyphenols influencing polysaccharide-polyphenol interaction

The various physical characteristics of polyphenols influence the affinity of polyphenols and the interaction between polysaccharides and polyphenols. An in-depth analysis of these factors is presented in the following sections.

### Molecular weight

The molecular weight of polyphenols plays a vital role in the interaction between polysaccharides and polyphenols. In general, polysaccharides are reported to have a remarkable ability to bind polyphenols with higher molecular weights (Bindon et al., 2010; Liu et al., 2020). A study conducted by Renard et al. (2001) concluded that the affinity of binding and interaction between apple cell wall polysaccharides and procyanidins (composed of (-)-epicatechin units) was more stable than that with the epicatechin monomers. This result was supported by other studies, which demonstrated that, along with molecular weight, the degree of polymerization also played a significant role in the interaction of polysaccharides and polyphenols. The interaction between the procyanidin and the cell wall material increases when the degree of polymerization of procyanidin also increases (Le Bourvellec et al., 2005; Liu et al., 2020).

A study by Liu et al., (2017a, 2017b) was conducted to measure the equilibrium adsorption of polyphenols (procyanidin B2, epicatechin, and phloridzin) on the surface of cellulose. The results were in the order of procyanidin B2> phloridzin>epicatechin. One possible explanation could be that the results reflect the number of aryl rings and ortho-phenolic groups in the polyphenol molecule. The aryl rings contribute to the hydrophobic interaction, and the ortho-phenolic groups participate in the hydrogen bonds between polyphenols and polysaccharides. In addition, an increase in the degree of polymerization makes the polyphenol molecule multidentate, which can interact with multiple molecules simultaneously, thereby increasing the interaction between the polyphenol molecule and the polysaccharide.

## Presence of functional groups in the polyphenol

The presence of functional groups such as hydroxyl, methyl, methoxy, and galloyl groups in the polyphenol structure significantly impacts the interaction of polyphenols and polysaccharides. The parameters, such as the number, position, and orientation of these functional groups in the aryl ring, significantly impact the interaction.

### Gallolyation

**Fig. 6:**
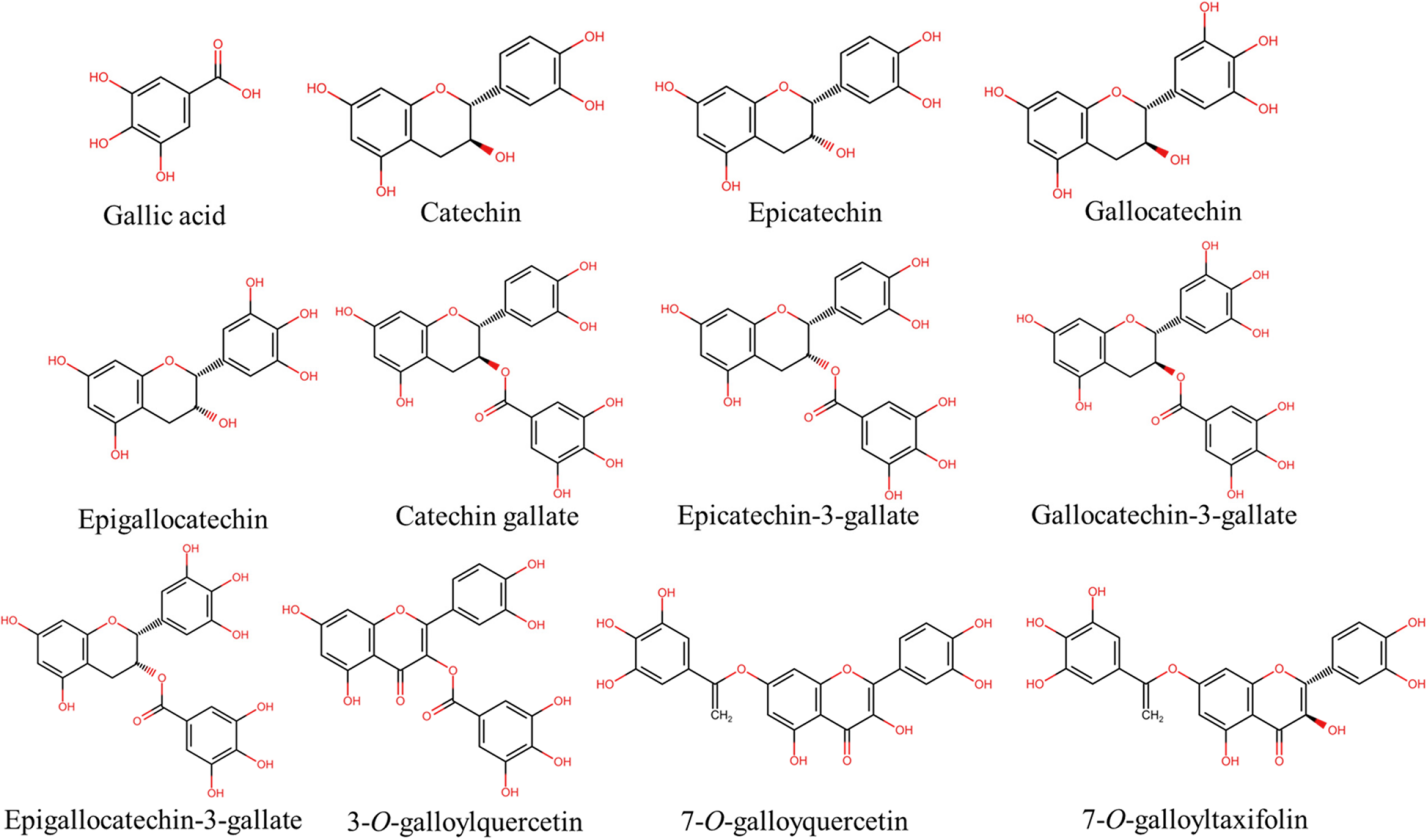
Chemical structures of selected galloylated phenolics (created using ChemSketch)

The addition of the galloyl group (radical derived from the removal of the hydroxyl group from the gallic acid) to phenolic compounds is known as the process of gallolyation (Fig. 6). Many studies suggest that the interaction between polysaccharides and polyphenols increases with the percentage of gallolyation (Liu et al., 2020; Mamet et al., 2018).

Tang et al. (2003) used 24 different polyphenols with cellulose and reported a positive correlation between the galloyl polyphenols and adsorption on the cellulose surface. It was reported that the conformational flexibility of the gallolyl group is restricted by the inter-galloyl C–C linkages, which are responsible for the increased hydrophobic interaction between the polyphenols and polysaccharides. A similar result was reported by Le Bourvellec et al. (2004); the increasing galloylation in procyanidin increased their interaction with the cell wall polysaccharide. When three (-)-epicatechin derivatives interacted with oat β-glucan, the interaction occurred in the following order: epigallocatechin-3-gallate > epicatechin-3-gallate > epicatechin (Wang et al., 2013). One of the possible explanations for this binding was the increase in the number of hydroxyl groups, galloyl groups, and aromatic groups, which enhanced the interaction through hydrogen bonding and hydrophobic interaction (Liu et al., 2020). Galloyl esters such as propyl gallate, lauryl gallate, and octyl gallate have a wide range of applications in cosmetic, food, and food packaging industries as they inhibit spoilage and rancidity (oxidative) development (Badhani et al., 2015; Karas et al., 2017).

#### Hydroxylation

**Fig. 7:**
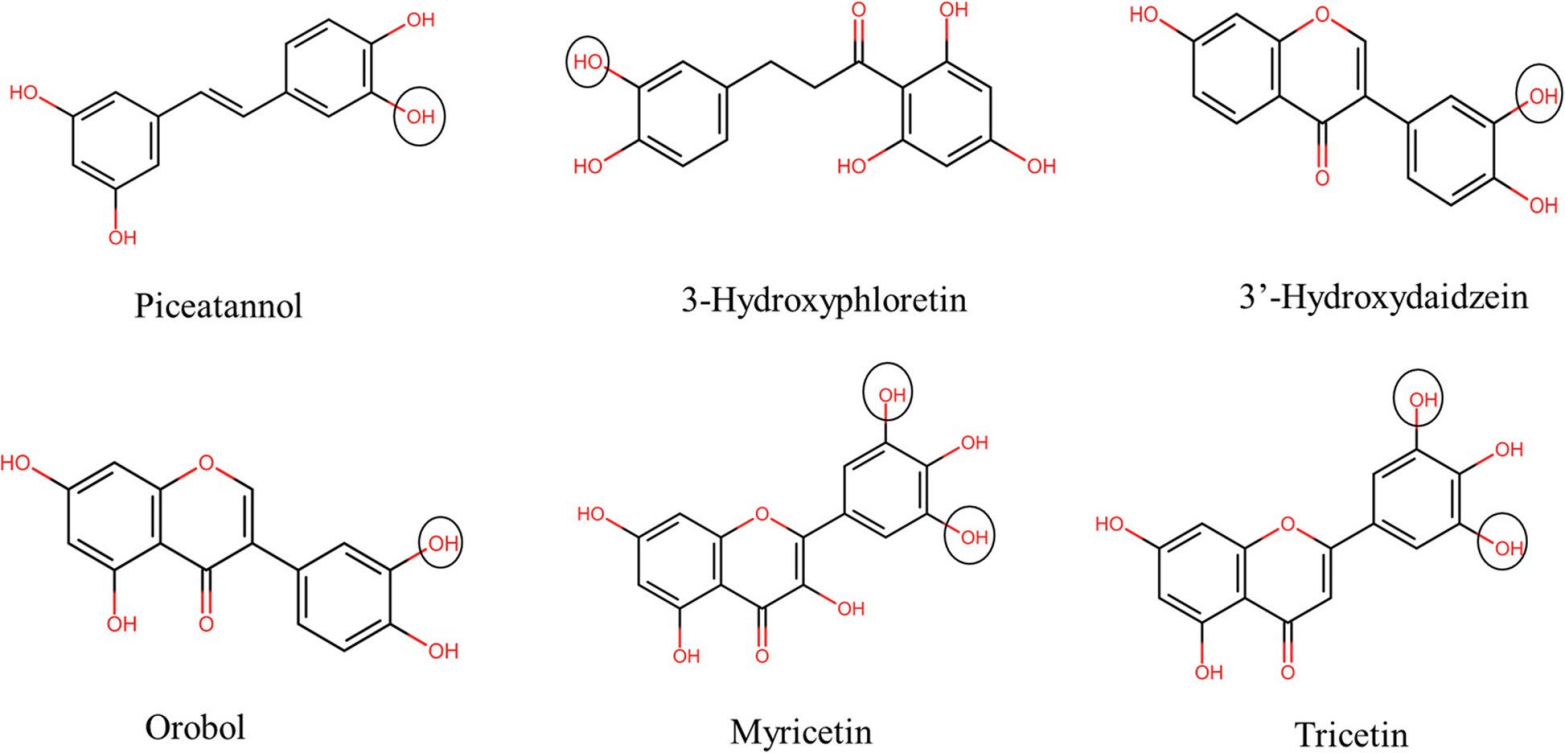
Chemical structures of selected polyphenols after hydroxylation through chemical or enzymatic modification (created using ChemSketch)

The addition of a hydroxyl group (-OH) to the polyphenolic compounds using chemical or enzymatic methods is known as hydroxylation (Fig. 7) (Lee et al., 2023). It shows a similar impact in the interaction, such as galloylation, the adsorption on the surface of polysaccharides increases with the presence of more hydroxyl groups in the structure of polyphenols (Liu et al., 2020). The presence of more hydroxyl groups in the B-ring of anthocyanin positively influenced the anthocyanin-pectin interaction (Buchweitz et al., 2013b).

In flavonoids, the interaction with polysaccharides was affected significantly by the degree of hydroxylation on the rings (A, B, and C) in the structure of flavonoids (Zhu et al., 2018). Wang et al. (2013) supported this observation and reported that the degree and location of the hydroxyl groups in the polyphenol structure have drastically affected the interaction between polysaccharides and polyphenols. The binding of flavonoids with oat β-glucan was in the following order: flavonol > flavone > flavanone > isoflavone. The adsorption on the surface of oat β-glucan increased upon the addition of a hydroxyl group on the B (apigenin to luteonin) and C (apigenin to kaempferol) rings. The hydroxylation effect was higher at the 3ʹ-position on the C ring than at the 3ʹ-position on the B ring. In addition to reducing adsorption capacity, the addition of hydroxyl groups to the C ring (from luteolin to quercetin) decreases its solubility.

A similar trend was observed in coumaric acid isomers with their interaction with oat β-glucan, the binding capacity increases in the order: *o*-coumaric acid (2-OH) > *m*-coumaric acid (3-OH) ≈ *p*-coumaric acid (4-OH) (Wang et al., 2013). Zhu et al. (2018) studied the interaction between arabinan-rich pectic polysaccharides (ARPP) and 27 phenolic compounds and determined that introducing a hydroxyl group enhanced the interaction. The study also revealed that hydrogen bonding acted as the primary driving force in the interaction. The number and position of the hydroxyl group in the polyphenol structure were essential parameters during the interaction of polyphenols and polysaccharides.

#### Methoxylation and methylation

**Fig. 8:**
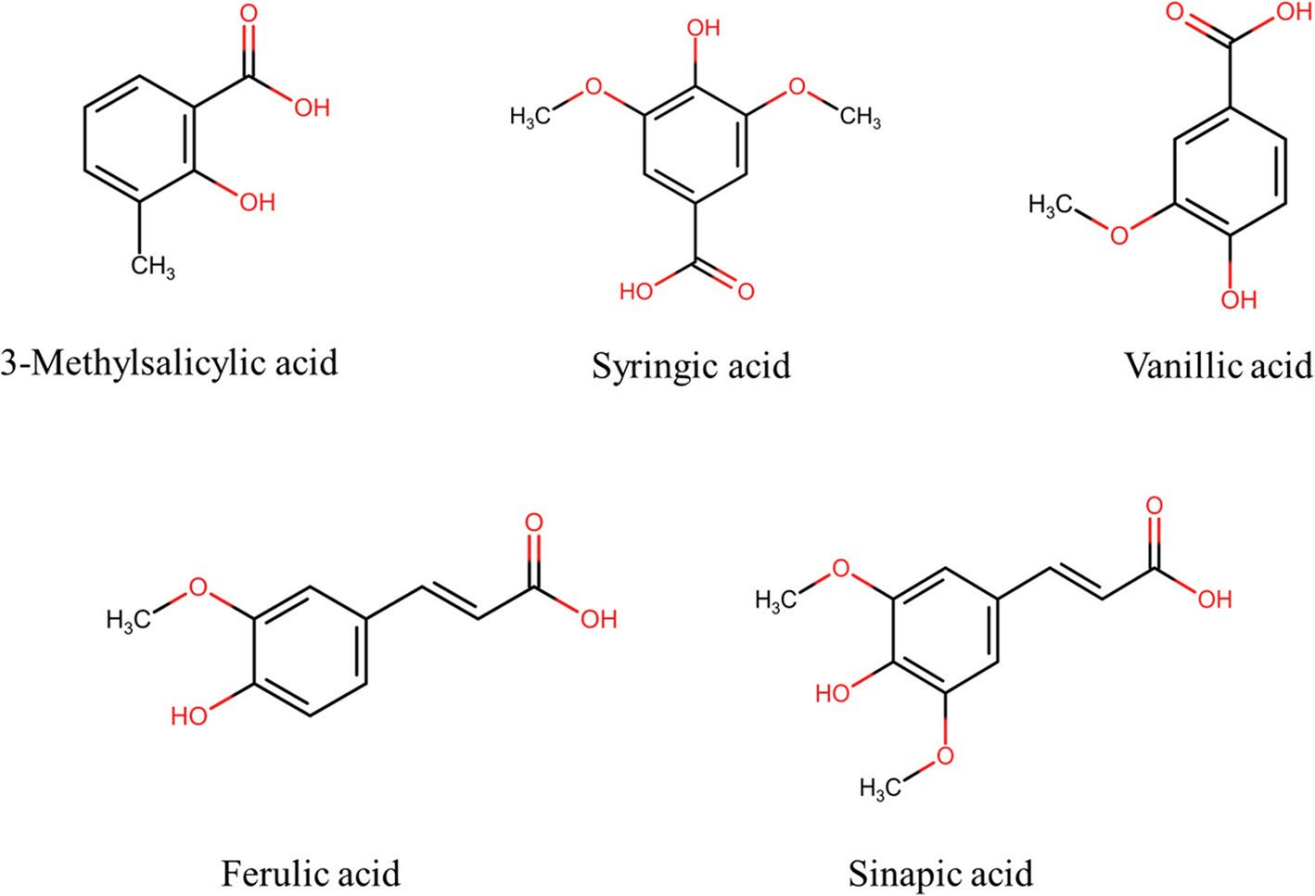
Chemical structures of selected methylated and methoxylated polyphenols (created using ChemSketch)

Methylation is a process of the addition of methyl group (-CH_3_) to the polyphenol molecule, and when methoxy group (-OCH_3_) is added to the polyphenol, then the process is known as methoxylation (Fig. 8). Like gallolyation and hydroxylation, adding methoxy or methyl groups to the polyphenol can either increase or decrease the interaction between polyphenol and polysaccharides. The response in the interaction depends entirely on the position of the addition and the change in the spatial structure of the polyphenol after the addition (Wang et al., 2013; Zhu et al., 2018).

The methylation and methoxylation did not increase the binding of flavones and hydroxybenzoic acid with oat β-glucan. However, structural changes occurred when the methoxy group was added to the B-ring of quercetin (a flavonol), and isohamnetin was formed, increasing the flavonol's binding capacity with the oat β-glucan. The solubility was also higher in isorhamnetin compared to that of quercetin (Wang et al., 2013). The binding capacity of hydroxybenzoic and hydroxycinnamic acids with ARPP decreased due to the methylation of these phenolics.

Whereas, when the methoxy group was added to the 5-position of the polyphenol, the adsorption of the polyphenol on the surface of the ARPP was increased, thus contradicting the results obtained by the addition of the methoxy group to the 3-position of the polyphenol (Zhu et al., 2018). It was also observed that ferulic acid (methylated caffeic acid) showed a higher binding capacity towards oat β-glucan than sinapic acid (methylated ferulic acid), which decreased the adsorption capacity, thereby reducing the interaction (Wang et al., 2013).

#### Glycosylation

The addition of a sugar molecule to the polyphenolic backbone is known as glycosylation, some polyphenols naturally possess a glucose group in their structure, which plays a significant role in their interaction with polysaccharides. The binding capacity of genistein was not affected by glycosylation, but daidzein and myricetin showed increased binding with oat β-glucan due to glycosylation. Polyphenols like naringin, rutin, and puerarin exhibited a decrease in their binding and interaction towards oat β-glucan compared to their unglycosylated form (Wang et al., 2013).

Conclusive results regarding the influence of the glycosyl group in the interaction of polysaccharides and polyphenols have not been reached in the literature. However, it can be suggested that the different degrees of glycosylation cause steric hindrance, ultimately resulting in a difference in the polysaccharide's adsorption rate. Chirug et al. (2018) reported that the binding and interaction between quercetin and pectin was more potent than that between rutin and pectin; this may be due to the presence of the disaccharide, thereby decreasing the number of binding sites for the flavonol and/or creating steric hindrance, restricting the interaction between these molecules.

## Methods to determine the interaction between polysaccharides and polyphenols

**Fig. 9:**
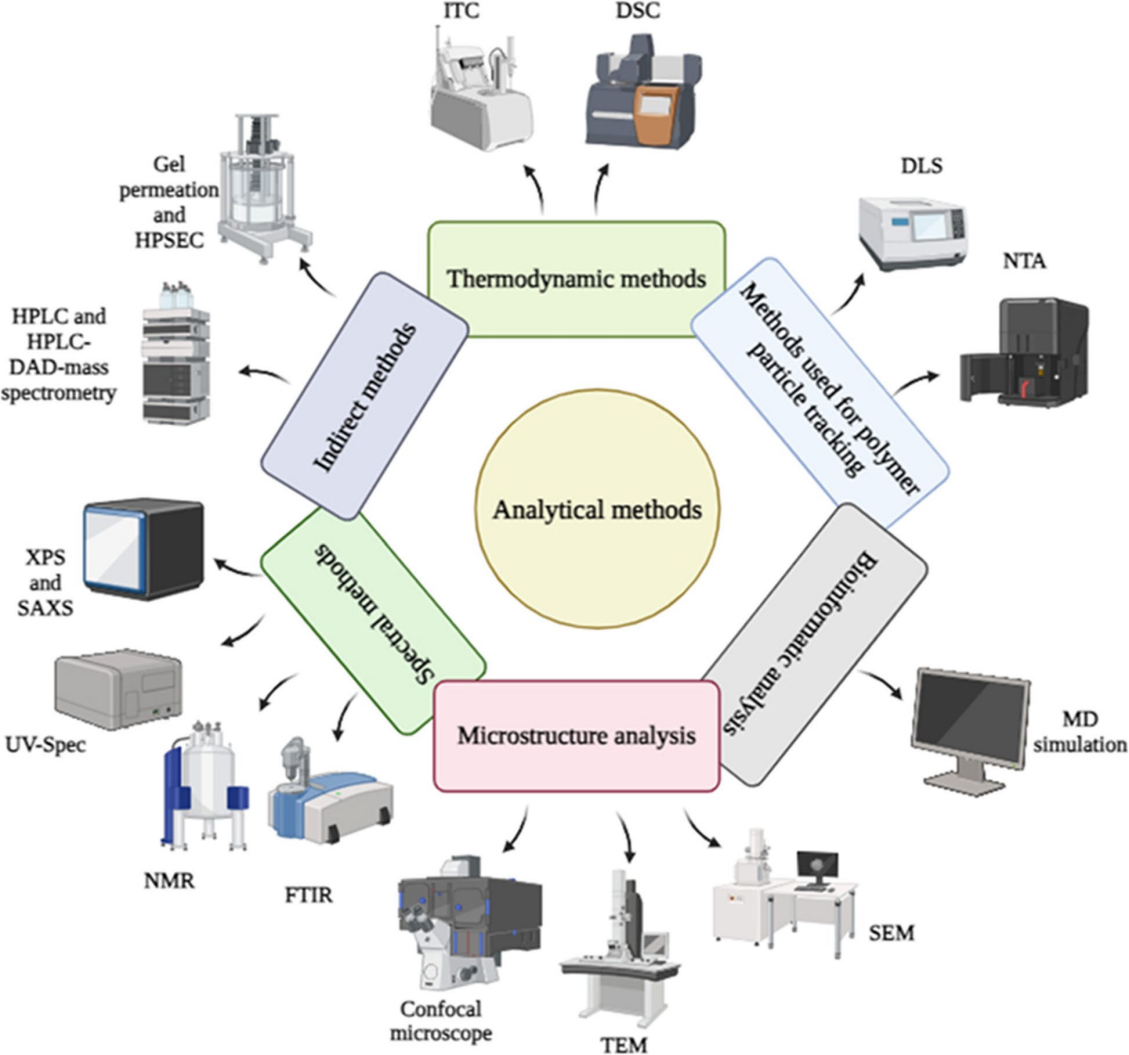
Different analytical methods for determining the interaction between polysaccharides and polyphenols (created using Biorender)

Numerous methods have been reported in the literature to determine the interaction between polyphenols and polysaccharides. Generally, the interaction between polysaccharides and polyphenols involves non-covalent binding (or conjugation) requiring low energy and highly reversible reactions. The analytical methods can be divided into categories based on their working principles, including spectral measurements, thermodynamic measurements, polymer particle tracking and its analysis, microstructure analysis, and indirect methods (Fig. 9).

### Spectral measurement

The spectral measurement of the interaction between polyphenols and polysaccharides is the primary and foundational method for determining their interactions. Various techniques are commonly employed, including UV-visible spectroscopy, FTIR, NMR, XPS, and SAXS.

#### UV–Visible spectroscopy

UV spectrometry (or UV-visible spectroscopy) relies on molecules'absorption of ultraviolet or visible light, which results in an electron shift within molecules. Recently, researchers have been using this technique to primarily analyze the interaction between polyphenols and polysaccharides (Bermúdez-Oria et al., 2020; He et al., 2019; Li et al., 2019a, 2019b; Mamet et al., 2018; Wei et al., 2019). The absorbance was used to detect and analyze the precipitate and cloud formation, indirectly indicating the formation of insoluble complexes among the macromolecules in the sample.

The advantages of this analytical method include its simplicity, speed, cost-effectiveness, and efficiency in detecting interactions. This method provides qualitative information about the solution's aggregate size and aggregation rate, providing insight into the relationship between structure and properties. Despite having several advantages, this method has a significant drawback as no direct information can be found about the binding sites, which lack specificity in the interaction between polyphenols and polysaccharides.

#### Fourier Transform Infrared (FTIR)

The information about the structure and dynamics of the polyphenol-polysaccharide interaction can be detected and analyzed with FTIR spectroscopy (Bermúdez-Oria et al., 2020; Brahem et al., 2019; Shi et al., 2019). The spectra of almost all polyphenols possess similar characteristic peaks that are attributed to -OH stretching and plane bending vibrations, C-C/C-C stretching vibrations, and C=C stretching vibrations in the aromatic ring (Guo et al., 2021). Upon interaction, researchers have observed changes in the characteristic peaks of the polyphenols and polysaccharides.

The presence of new peaks in the spectrum was observed for the covalently linked polysaccharide-polyphenol interaction (Ahn et al., 2017; Moreno-Vásquez et al., 2017; Wei & Gao, 2016; Zhou et al., 2020). Wei and Gao (2016) reported that the chitosan-chlorogenic acid complex had a similar FTIR spectrum to that of the chitosan. However, peaks at 1,730 and 1,640 cm^−1^ are attributed to the C=O stretching in the esters (which connects the -OH group) and C=O stretching in the chitosan (which connects the amide group), respectively.

Principal component analysis (PCA) can also be used in studies to identify the specific wavelength of the polyphenols and the polysaccharides (Brahem et al., 2019). Shi et al. (2019) utilized PCA to differentiate between samples of puree and pomace, both with and without epigallocatechin gallate (EGCG). Through infrared spectra, the study quantitatively discerned the presence of polyphenols or polysaccharides within the complex without altering the signals, such as the absence of specific peak modifications or shifts for either the polysaccharide or the polyphenol.

Consequently, tracking variations in the intensity of polysaccharide/polyphenol-specific vibrations uses different spectra. Additionally, their analysis suggests that interaction between CPS and polyphenols has minimal impact on molecular structure at the infrared scale investigated, specifically on covalent bonds. Therefore, FTIR can detect the presence of a complex in the sample by analyzing the peak shift, peak intensity, and the presence of a new peak compared with the control polysaccharide or polyphenol sample. The major advantage of the technique is that it is simple, fast, non-destructive, and highly sensitive. Though FTIR sheds light on the molecules in the complex, no information about the interaction mechanism could be obtained.

#### Nuclear magnetic resonance (NMR)

The NMR technique has significantly contributed to exploring interactions, starting with initial low-resolution studies, which primarily focus on moisture content, and moving up to high-resolution investigations involving diverse liquid and solid matrices (Koshani et al., 2020). NMR spectroscopy in solution offers precise insights into the fundamental structure of a sample. Moreover, it is a valuable tool for exploring the mechanisms underlying interactions.

When a polysaccharide interacts with a polyphenol, this interaction may change the chemical shifts of hydrogen or carbon atoms. These alterations can stem directly from the interaction or indirectly from conformational changes induced by binding. The chemical shift is unique to individual carbon or hydrogen atoms on a polysaccharide or polyphenol, enabling precise binding site identification. This specificity aids in determining the mechanism of interaction with accuracy (Bermúdez-Oria et al., 2020; Fernandes et al., 2020; Liu et al., 2020).

Numerous studies have detailed the significance of NMR analysis in providing information about the interaction among polysaccharides and polyphenols. Specifically, the hydrophobic interaction between cyclodextrin (CD) and polyphenols was analyzed, where the chemical shifts of the H-3 and H-5 protons of CD were observed, which reflects the interaction of the polyphenol within the cavity of CD (Cai et al., 1990; Connors, 1997). This method can subsequently ascertain the structure of the complex: if only H-3 of CD is displaced during the interactions, it indicates shallow penetration of the polyphenol into the CD cavity; however, if H-5 is also shifted, it suggests that the molecule is entirely encompassed within the cavity (Connors, 1997).

Polyphenol-polysaccharide conjugates can also be characterized using solid-state NMR, such as ^13^C NMR. The signals were seen at C-1, C-2, C-3, C-4, C-5, C-6, and in the ^13^C NMR spectra of chitosan. Compared to chitosan, phenolic acid-chitosan conjugates show extra peaks between 110 and 150 ppm, attributed to the phenolic groups C = C double bond (Liu et al. 2014). The formation of carbonyl (C = O) groups between the amino groups of chitosan and the carboxyl groups of phenolic acid is responsible for the increased signal at 174.8 ppm (Rui et al., 2017). A new peak at 163 ppm was also observed in the spectrum due to forming an imine (C = N) group within the complex (Saranya et al., 2018).

Apart from the 1D NMR (^1^H and ^13^C), 2D NMR techniques such as total correlation spectroscopy (TOCSY), homonuclear correlation spectroscopy (COSY), heteronuclear single quantum coherence (HSQC), and heteronuclear multiple quantum correlation (HMBC) spectroscopy are widely used for the analysis of polyphenols (Rouger et al., 2019), polysaccharides (Guo et al., 2021) and the polyphenol-polysaccharide complex (Pawlaczyk-Graja, 2018). Thus, it can be noted that NMR plays a crucial role in revealing specific details about the polyphenol-polysaccharide complex, such as the degree of substitution, stoichiometry, affinity, binding site, and molecular structure. A few drawbacks observed in the techniques include the need for highly pure (homologous) samples, which work well for small molecules.

#### X-ray photoelectron spectroscopy (XPS)

The XPS determines the empirical formula, elemental composition, chemical, and electronic states of elements in samples (Hollander & Jolly, 1970). Carbon, hydrogen, and oxygen are the fundamental components of polysaccharides and polyphenols molecules, with polysaccharides having greater oxygen-to-carbon ratios. Post interaction, more polyphenol is adsorbed onto polysaccharides, which lowers the O/C ratio. For instance, Wei et al. (2019) suggested a higher mass ratio of tannic acid (TA) to wheat starch (WS) was associated with a decrease in the O/C ratio. Further scanning electron microscopy (SEM) analysis showed three stages of the interaction process: TA gradually adsorbs onto WS aggregate surfaces to form a thicker TA molecular layer, and finally reaggregates to form larger aggregates or even precipitates. This observation coincides with the study by Pascal et al. (2007) which analyzed the interaction of the flavan-3-ols and proline-rich proteins.

The major advantage of this method is that it is non-destructive, surface-sensitive, and provides insights into the chemical bonds and elements within the sample. Interestingly, no research has been published using XPS to identify changes in the O/C ratio following polysaccharide-polyphenol interaction, suggesting that future studies could clarify element ratio changes. One of the well-known drawbacks of XPS is that, though it can identify the elements within the complex and their ratios, it cannot provide details about the binding site within the molecule.

#### Small-angle X-ray scattering (SAXS)

The SAXS can analyze geometric features and structural details, including size distribution, particle shape, and domain arrangement of the particles. Furthermore, it can reveal the interaction among macromolecules dissolved in solution (Muroga et al., 1987). SAXS is generally used to analyze the interaction between polyphenols and proteins (Jöbstl et al., 2004; Shi et al., 2017). However, only a few studies have reported the interaction of polysaccharides and polyphenols.

Carn et al. (2012) reported that procyanidins with DP>10 tend to mix with the polysaccharide hyaluronan (HA) to produce a variety of three-dimensional (3D) aggregates using SAXS with synchrotron radiation. Procyanidins DP<10, on the other hand, typically combine with HA to create low-density oligomers. This suggests that procyanidin chain length determines the structure and molecular weight of polyphenol-polysaccharide complexes (procyanidin-HA complex), irrespective of procyanidin concentration; shorter procyanidins form coil-like aggregates, while longer ones form larger bushy aggregates or microgels with HA. Due to the need for synchrotron radiation, this approach has not been used much. However, it can potentially help reveal information on the structure and conformation of the complex.

The primary advantage of using synchrotron radiation scanning technology is its exceptionally high incident optical density, which can be used to track the dynamic processes of structural changes at the nanoscale, reliably analyze the nanostructure of samples, reduce the size of the light spot, and increase the signal-to-noise ratio. This technology provides quick results, but its interpretation requires complex data analysis. This limits the use of this technique to the complexes involving macromolecules.

### Thermodynamic method

The thermodynamic changes in the macromolecules during complex formation can be determined using two established techniques: differential scanning calorimetry (DSC) and isothermal titration calorimetry (ITC).

#### Differential scanning calorimetry (DSC)

The DSC is an effective analytical method for evaluating the thermal characteristics of the complex, such as enthalpy change (ΔH) and denaturation temperature (T_d_), which includes onset (T_o_), peak (T_p_), and conclusion (T_c_) temperatures. The values of T_d_ and ΔH offer information about the conformation and heat stability of the samples, respectively (Höhne et al., 2013).

The interaction of polysaccharides with polyphenols has been less frequently explored using this technique than with other macromolecules such as proteins and lipids. One such study to analyze the polysaccharide-polyphenol interaction using DSC was conducted by Guo et al. (2018), who found that the denaturation temperature (T_d_) and enthalpy change (ΔH) values of corn silk polysaccharide-flavonoid complex (CSP-CSF complex) were notably elevated compared to those of unbound CSP. This suggests that the CSP-CSF complex exhibits enhanced stability and a more organized structure than the polysaccharides (without flavonoids). In their examination of various maize starches, Liu et al. (2017a, 2017b) observed that the introduction of low concentrations of oligomeric procyanidins (OPCs) inhibited the retrogradation process in amylose maize (HAM), normal maize (NM), and amylopectin maize (APM) starches.

Further analysis of the DSC curves unveiled distinct trends in the ΔH value among NM-OPC, APM-OPC, and HAM-OPC complexes. In this context, the ΔH value indicates the orientation and stacking energy of the double helix within the crystalline and amorphous regions of starch granules. Another interesting observation in this research was that the OPC hydroxyl groups interact with the starch hydroxyl groups, ultimately forming the hydrogen bonds (which leads to modification of the starch polymer). The OPCs inhibit starch retrogradation in a concentration-dependent manner (Liu et al., 2017a, 2017b).

The major advantages of this method are that it requires fewer samples, rapid detection, high sensitivity, and reliable results (Bermúdez-Oria et al., 2020; Liu et al., 2020). The disadvantages of the technique include a lack of technical details about the molecular properties of the sample and minor changes in the parameters of the sample, such as size, weight, and heating rate, which affect the results.

#### Isothermal titration calorimetry (ITC)

The ITC is an effective technique widely used to determine endothermic and exothermic thermodynamic changes associated with creating complex interactions involving natural products such as proteins, polysaccharides, and polyphenols. Significantly, it can provide a thorough kinetic profile within a single experimental design (Callies & Hernández Daranas, 2016). This technique provides direct measurements of the stoichiometry (n) and binding constant (K_av_). It also provides indirect measurements of thermodynamic parameters such as entropy (∆S) and free energy (∆G) of binding (Brahem et al., 2019; Liu et al., 2020). Data obtained from ITC experiments can be utilized to calculate Gibbs'binding energy using Equation 1-

1 $$\Delta\mathrm G=-\text{RT}\ast\text{lnKav}$$

where R represents the ideal gas constant and T signifies absolute temperature.

Furthermore, the change in entropy can be evaluated using the relationship:

2 $$\triangle\mathrm G=\triangle\mathrm H-\;\mathrm T\triangle\mathrm S$$

From a thermodynamic standpoint, ΔG encompasses two distinct contributions (ΔS and ΔH), collectively reflecting the binding strength. The release of water molecules and ions, hydrophobic interaction, and conformational changes are generally associated with ΔS. Conversely, van der Waals forces, electrostatic interaction, protonation, and hydrogen bonding are associated with the ΔH (Callies & Hernández Daranas, 2016).

Hydrophobic interaction, being a long-range phenomenon, relies on the solubilities of CPSs and polyphenols in the solvent. These interactions are derived from the displacement of stable water molecules from the binding pocket upon complexation. In contrast, hydrogen bonds are considered short-range phenomena and typically enhance the complex's stability (Israelachvili, 2011). Various combinations of ΔH and ΔS can generally result in the same binding affinity, i.e., the same ΔG, and thus the same binding constant (K_av_).

Consequently, even if the binding affinity remains constant, the behavior and response of polyphenols to environmental changes or CPS adsorption could vary, along with the enthalpy or entropy components (Liu et al., 2020). Therefore, both hydrogen bonding and hydrophobic interactions between the polysaccharide-polyphenols complex can be revealed using ITC (Le Bourvellec et al., 2004, 2005). Research conducted on pear cells revealed that the binding constant (Kav) for interaction between pear cell walls and procyanidins (DP: 26) was approximately on the order of 102 M^−1^ (Brahem et al., 2019).

Conversely, interaction between pectins (including both commercial pectins and the hairy regions of pectins) and procyanidins exhibited association constants ranging between 102 M^−1^ and 104 M^−1^ (Le Bourvellec et al., 2012; Watrelot et al., 2013). Additionally, the affinity constants for interaction between procyanidins and sugar beet arabinan and debranched arabinan were calculated as 391 M^−1^ and 540 M^−1^, respectively (Fernandes et al., 2020). Generally, an association constant exceeding 104 M^−1^ indicates high affinity (Turnbull & Daranas, 2003).

One of the major advantages of the ITC is that it provides direct insights into the thermodynamic information about the interaction. Furthermore, it does not require the samples to be optically transparent. There are a few drawbacks associated with this technique, as it is time-consuming and susceptible to impurities, so the samples must be contaminant-free.

### Polymer particle tracking and analysis

The change in the size distribution among the particles during the polyphenol-polysaccharide interaction has been analyzed using dynamic light scattering (DLS) and nanoparticle tracking analysis (NTA).

#### Dynamic light scattering (DLS)

The DLS is a precipitation-based method similar to turbidimetry. Aggregates may produce insoluble complexes as they become more significant in size, scattering light, and eventually precipitating. DLS can detect particle size or diameter variations, usually in the submicron range. Nevertheless, dust particles must be removed from the solution for DLS to function properly, as their existence can significantly affect the accuracy of the findings. Very little literature has been done on the interaction between polysaccharides and polyphenols.

Carn et al. (2012) analyzed the hyaluronan-tannin complex using DLS and found that these macromolecules form complexes with diameters up to 25 nm and large particles (up to 10 times bigger). The intensity of the complex formation was directly proportional to the amount of tannins present in the sample. Another study by He et al. (2019) determined the DLS of the konjac glucomannan (KGM) polysaccharide and tannin (TA). This study also found a similar result: a high polydispersity index (PDI) was observed in the KGM and decreased upon complex formation. The presence of pectin (citrus) decreased tannin's PDI, which might indicate the presence of the homogeneous aggregate in the solution (Mamet et al., 2018).

The technique is rapid and requires a minimal amount of samples. The results were accurate for the monodisperse samples but not applicable for the polydisperse samples (the peak resolution would be low), so dust particles or other contaminants may interfere with the results.

#### Nanoparticle tracking analysis (NTA)

Highly accurate findings can be obtained by using NTA (Nanoparticle Tracking Analysis), which tracks the Brownian motion of each particle that has been discovered and analyzes it separately to determine its hydrodynamic diameter. It is generally combined with the DLS method for aggregate characterization. NTA usually provides better resolution; however, DLS allows quick evaluation of average size and polydispersity.

Furthermore, NTA can be used to track the light-scattering intensity of each particle to detect the presence of aggregates, even in the absence of appreciable changes in particle size (Liu et al., 2020). Changes in the size distribution of colloids caused by aggregation between commercial polysaccharide supplements (mannoprotein [MP] or gum Arabic [GA]) and grape seed tannins in wine have been assessed using NTA. GA exhibited relatively weak interaction with tannins, while MP produced highly light-scattering aggregates upon such interaction. The size analysis for monodisperse and polydisperse samples was more accurate, resulting in better peak resolution than DLS (Li et al., 2019a, 2019b). One of the significant drawbacks of this technique is that it is time-consuming, requires special software skills, and may not always be reproducible.

### Microstructure analysis

The structural (microstructural) analysis of the macromolecules after their interaction can be analyzed using a scanning electron microscope (SEM), transmission electron microscope (TEM), and confocal laser scanning microscope (CLSM).

#### Scanning electron microscope (SEM)

In scanning electron microscopy (SEM), an electron beam is used to scan the surface of a sample, yielding its surface topography with a resolution of <1 nm (Goldstein et al., 2018). Many studies have used this analysis to predict the macromolecule's structural level alterations after interacting with other molecules. However, the majority of the studies have reported the surface topography of the polysaccharide-polyphenol complex, and no information regarding the complex can be obtained; one of the possible reasons stated is that the polyphenol molecule becomes unstable, which makes the observation difficult (Liu et al., 2020).

A study reported the topographical changes in the tannic acid-wheat starch complex, which was initially spherical. However, as the mass ratio of tannic acid to wheat starch increased, the complex grew and finally formed a micelle. It was also stated that tannic acid formed hydrogen bonds with wheat starch and was absorbed in a monolayer, eventually forming a complex structure (Wei et al., 2019).

The major advantage of this technique is that it produces a three-dimensional image of the complex structure, and the sample preparation is simple (Guo et al., 2018). However, the technique is limited to only solid samples.

#### Transmission electron microscopy (TEM)

Transmission electron microscopy (TEM) is a powerful tool for examining the organization, structural, and morphological properties of materials since it has higher resolution and magnification than scanning electron microscopy (SEM) (Reimer, 1984).

However, since the electron beam must pass through the material, TEM applications require carefully prepared tiny samples, usually thinner than 100 nm. The final result shows the sample projected in two dimensions. Only a few studies offer in-depth insights into the structural morphology of the polysaccharide-polyphenol complex.

A study by He et al. (2019), combined the TEM with the DLS analysis and concluded that the tannic acid-konjac glucomannan (KGM) complex initially formed aggregates, eventually developing into a complex. After their interaction, Patel et al. (2011) observed a similar aggregate structure of epigallocatechin gallate (EGCG) and methylcellulose.

There are many advantages of using TEM for the structural characterization of the polysaccharide-polyphenol complex, including powerful magnification and structure resolution. The obtained images can also be interpreted for the chemical information within the structure (He et al., 2019; Mamet et al., 2018). Major drawbacks of the method are preparing samples smaller than 100 nm and the limited availability of electron-transparent materials (which allow electrons to pass through with minimal scattering), which limits its application to macromolecular structural analysis.

#### Confocal laser scanning microscopy (CLSM)

The CLSM uses a laser beam to capture a focused image at a specified depth, achieving a resolution at the submicron level. The resulting image can depict either a two-dimensional structure or a reconstructed representation of a three-dimensional structure (Hutzler et al., 1998). For the CLSM analysis, the sample whose position has to be determined, for example, polyphenol molecules were treated with fluorescent dyes, and the polysaccharide molecules were labeled using fluorescent tags. For the visualization of the polyphenols (flavonoids), 2-aminoethyl-diphenylborinate was used for the amplification of the fluorescence signal (Liu et al., 2019a, 2019b, 2019c). As a result of this experiment, direct evidence of the adsorption mechanism of polyphenols on the polysaccharide surface was observed (Liu et al., 2020; Padayachee et al., 2012b).

The major advantage of CLSM is that it is non-invasive and can be used to analyze live cells. The technique has several disadvantages, including the coherence of laser light and its fluctuations that may induce artifacts, while significant photobleaching can occur (Liu et al., 2020). Additionally, scanning is often slow and constrained in dynamic tracking studies, and lasers can potentially harm living cells. Furthermore, the resolution tends to be lower compared to camera-based detection methods.

### Bioinformatic analysis

Molecular dynamic simulation is a common technique used for the bioinformatic analysis of the polyphenol-polysaccharide complex. Molecular dynamics (MD) simulation is a computational approach that allows the prediction of the dynamic evolution of a system comprising interacting molecules, utilizing Newton’s laws. In addition to uncovering allosteric or cryptic binding sites, the simulation technique can directly predict small molecules'binding energies (Fernandes et al., 2014; Liu et al., 2020, 2018a, 2018b, 2018c).

Fernandes et al. (2016) utilized MD simulation to explore the mechanisms of copigmentation, by using the models of catechin, oenin, and pectin in aqueous solution. The pectin polysaccharides were simplified using a 16-monosaccharide fragment with limited methylation and acetylation. In the oenin–pectin model simulations, the sugar molecules exhibited robust interaction and enveloped the anthocyanins on both sides. In the more intricate simulation involving the polyphenols, the distance between the catechin atom and the pectin molecule to the C2 atom of oenin was approximately 3.0 Å. This proximity hindered the entry of solvent molecules and interaction with oenin, elucidating the enhanced stability of the ternary complex. However, clear insights into the macromolecule interaction could be obtained using a combination of MD and other simulation techniques (Monte Carlo simulation, molecular docking, etc) (Chen et al., 2019).

Only limited studies have focused on the MD simulation analysis of the polysaccharide-polyphenol interaction, this could be due to the lack of availability of the 3D structure of the macromolecules. The well-known three-dimensional structure database for carbohydrates is Gluco3D (Pérez et al., 2015), and databases for the polyphenols include PubChem (Kim et al., 2019a, 2019b) and ChEMBL (Gaulton et al., 2017).

The major advantage of this technique is that it removes the limitations of the experimental conditions and can verify the laboratory experiment results. However, the MD simulation consumes more time and requires a sophisticated computer system.

### Indirect methods for analysis

A complex is indirectly characterized by analyzing the molecules that react with it (polysaccharides and polyphenols) instead of focusing on the complex itself. The structural characterization of the macromolecules may indirectly provide information about the impact of interaction on their morphology. These analyses can be conducted using HPLC-DAD, chromatography techniques for polyphenols, and HPSEC for the polysaccharides (Liu et al., 2020).

Through indirect techniques, it is feasible to discern the correlations between the structure and properties of polyphenols and polysaccharides, which allows calculating the number of polyphenols bound to each polysaccharide molecule. Adsorption phenomena can be characterized by employing the Langmuir isotherm, which aids in determining binding parameters such as the apparent affinity constant (K_L_) and the total quantity of available binding sites or apparent saturation level (Nmax) (typically expressed in grams per gram of adsorbent) (Bindon et al., 2012; Le Bourvellec et al., 2004).

Some studies have also employed pseudo-first and second-order reaction, Weber-Morris (interparticle) diffusion models to evaluate the adsorption mechanism of the catechins on the surface of cellulose/pectins. It was observed that the initial adsorption of catechin on the surface of pectin was faster than that of cellulose. However, in the later stages, the adsorption slowed down in both the polysaccharide molecules (Liu et al., 2019a, 2019b, 2019c). These indirect methods provide insights into the changes in the physical characteristics of the polysaccharides upon interaction with the polyphenols. Few studies have reported that the interaction of polyphenols decreased the polysaccharide's viscosity and pseudoplastic properties (Tudorache & Bordenave, 2019; Tudorache et al., 2020).

**Fig. 10:**
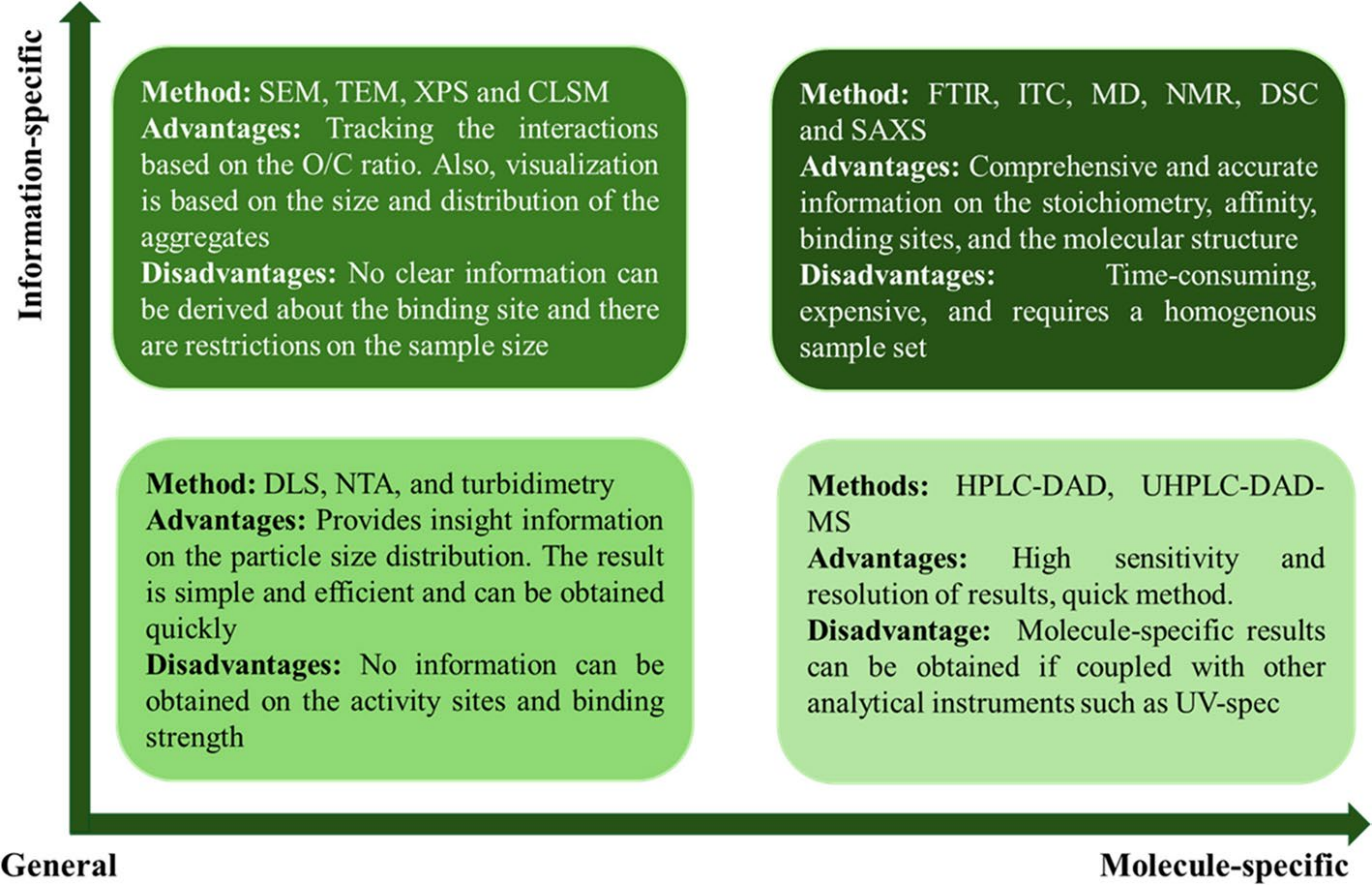
Classification of methods used to detect the interaction between the polysaccharide and polyphenol (created using Microsoft PowerPoint)

In summary, several analytical techniques have been used to analyze the interactions of the polysaccharide-polyphenol complexes with technique-specific advantages and disadvantages (Fig. 10). Spectral methods such as ultraviolet-visible spectroscopy (UV-Vis), Fourier transform infrared spectroscopy (FTIR), nuclear magnetic resonance (NMR), X-ray photoelectron spectroscopy (XPS), and small angle X-ray scattering (SAXS) reveal an in-depth molecular change upon interaction within the complex. In contrast, thermodynamic methods such as differential scanning calorimetry (DSC) and isothermal titration calorimetry (ITC) provide details about binding affinities and energy changes when interactions occur. The particle tracking methods such as dynamic light scattering (DLS) and nanoparticle tracking analysis (NTA) provide information about the particle size distribution and methods such as scanning electron microscopy (SEM), transmission electron microscopy (TEM), and confocal laser scanning microscopy (CLSM) (microscopic analysis) aid in visualizing the interaction-induced changes in the structure of the molecules. Computational techniques such as MD simulations help predict the interaction mechanisms, while indirect techniques such as chromatography assist in understanding and characterizing the binding-induced changes in the structure of the molecules. Since each method has advantages and disadvantages, it is up to the researcher to decide which technique to use based on the study's objectives and the level of molecular detail required.

## Application of the polyphenol-polysaccharide complex in the food industry

The polysaccharide-polyphenol complex can serve as the best alternative for biodegradable packaging and as a preservative in the food industry. This complex has been explored in numerous studies as a packaging film as well as a food preservative (Jancikova et al., 2019; Moreno et al., 2020; Riaz et al., 2020; Rojas-Bravo et al., 2019; Romani et al., 2018; Sun et al., 2017, 2018). Polysaccharides such as cellulose, starch, and pectin have been intensively studied as an eco-friendly alternative for food packaging for the last few decades (Riaz et al., 2018; Ye et al., 2018).

Recently, researchers have fortified these biodegradable food packaging materials with polyphenols to extend the shelf life of food. For instance, grass carp fillet was wrapped using chitosan film coated with polyphenols (obtained from thin apple slices). It was observed that the fillets stored in this packaging material showed decreased protein and lipid oxidation. In addition, it was also observed that there was a change in the color, pH, and amino acid contents and a significant reduction in microbial proliferation and water-holding capacity. Based on these observations, it was concluded that the fortified packaging was responsible for the favorable food packaging properties (water retention, antimicrobial, and antioxidant properties) (Sun et al., 2017, 2018). Guo et al. (2020) studied the effect of combining potato starch and sea buckthorn pomace extract and used it as a packaging film for beef jerky products. The major outcome of the research was that the film prevented quality loss of the beef jerky by reducing water loss and total nitrogen content (volatile), maintaining the meat's color, and acting as an antimicrobial agent.

Fortified packaging has not only been explored in meat, but researchers have studied its potential in plant-based food as well (Moreno et al., 2020; Riaz et al., 2018; Rojas-Bravo et al., 2019; Romani et al., 2018; Wu et al., 2019). Romani et al. (2018) observed that apple slices dipped in the coating material containing rice starch combined with pink pepper extract significantly reduced oxidation, which caused browning in the apple. Similar studies were also conducted in oils, Riaz et al. (2020) added soybean oil to the packaging film containing lime peel pectin and polyphenols; the oxidation was delayed for 30 days at 27 ºC under fluorescent light. The amount of thiobarbituric acid reactive substances (TBARS) in the oil was decreased, ultimately delaying its oxidation and extending its storage life.

Researchers have also explored this fortified packaging as an innovative and intelligent packaging. The smart or intelligent packaging is pH dependent; these films could be used as a freshness indicator for various food products. When k-carrageenan film was fortified with curcumin (yellow), it turned red upon subjecting it to alkaline conditions (Liu et al., 2018a, 2018b, 2018c). This film was used as a freshness indicator in animal products such as shrimp and pork. Mulberry extract containing polyphenols was added to the k-carrageenan film to detect milk spoilage (Liu et al., 2019a, 2019b, 2019c). Further research could shed light on the specific combination of intelligent packaging films that can be obtained, which could benefit consumers.

Recent studies have revealed the preservative nature of the polyphenol-polysaccharide complexes. Xue et al. (2024) reported that the chitosan-tea polyphenol complex enhanced the preservative effect on strawberries, suggesting its potential use for other fruits and vegetables. This effect is attributed to the formation of chitosan films, which reduce gas exchange on the fruit surface and regulate respiration. Kanokpanont et al. (2018) grafted three phenolic acids—gallic acid, caffeic acid, and chlorogenic acid—onto chitosan to evaluate its effectiveness as a preservative for Japanese sea bass (*Lateolabrax japonicus*) fillets. The study revealed that the chitosan-phenolic acid complex inhibited bacterial growth and scavenged excess free radicals, demonstrating its antioxidant capacity.

Fresh-cut apples coated with chitosan incorporated with guava leaf flavonoids exhibited improved quality in terms of firmness, browning index, and weight loss. The coating also enhanced the apples'nutritional value, increased antioxidant activity, and protected against bacterial contamination (Wang et al., 2023a, 2023b). This study further confirmed the potential of polyphenol-polysaccharide complexes as effective preservatives in the food industry.

Beyond their use as preservatives, these complexes are extensively studied for their potential to regulate glucose metabolism. When ferulic acid or caffeic acid combines with starch, it forms a complex that lowers blood glucose levels by promoting resistant starch formation and intercepting phenolic substances. Additionally, these complexes enhance the bioavailability of polyphenols, suggesting a promising approach for the starch-based food industry to incorporate polyphenols and regulate starch metabolism for better blood glucose control (Li et al., 2020).

A study on cassava starch and epigallocatechin-3-gallate (EGCG) revealed reduced glucose digestion and release rate from the starch. The complex improved cassava starch's relative crystallinity and thermal stability, which could pave the way for developing the starch-based delivery system in food processing industries (Zhu et al., 2021). Similar results were observed when potato starch was combined with phenolic acids such as tannic acid, naringin, and protocatechuic acid. This combination enhanced the starch’s antioxidant activity and improved its stability by altering its rheological properties (Chen et al., 2023).

Limited studies have explored polyphenol-polysaccharide complexes as delivery systems to enhance the stability and bioavailability of polyphenols in food systems. Additionally, these complexes serve as encapsulant matrices, protecting polyphenols during digestion and improving their bioavailability, as polyphenols are inherently unstable bioactive compounds. When curcumin was coated with gum nanoparticles and examined in a high-salt food system, its stability remained above 80% even at high NaCl concentrations, indicating strong salt resistance and the potential for enhanced bioavailability (Ai et al., 2023).

Beyond food packaging and preservatives, polyphenol also plays a prominent role in the astringency of wine. The astringency is a vital factor when assessing wine's sensory attributes. It arises from concentrated tannins and saliva proteins interaction, which leads to aggregate formation and precipitation (Guo et al., 2022; Quijada-Morín et al., 2014; Watrelot et al., 2017). The perception of astringency can vary depending on the interaction of tannin with other macromolecules such as proteins, polysaccharides, and lipids.

The polysaccharides are found to be competing with the salivary proteins for the tannin (concentrated) substrate, which inhibits the tannin-protein interaction that leads to the reduction of the astringency of wine (Quijada-Morín et al., 2014; Watrelot et al., 2017). Notably, the structure of polysaccharides was related to the relationships between polysaccharides and tannins. The non-covalent bonding (or conjugation) of tannins was regulated by various factors, including the composition of monosaccharides, the degree of methylation in homogalacturonans within pectin, and the linkage patterns in the hairy region of pectin (Guo et al., 2022).

Polyphenol-polysaccharide complexes offer synergistic benefits by slowing polysaccharide digestion, thereby inhibiting blood glucose spikes. Polyphenols embedded within the polysaccharide matrix are also protected during digestion, allowing for controlled release in the gastrointestinal system. Therefore, these complexes and their interaction have a wide range of applications in the food processing industry, including preservatives, biodegradable packaging films, delivery systems, and modification of starch-based products. Based on the researcher's objective, they can also be used as a potential functional food.

## Conclusion and future perspective

The crucial application of the polyphenol-polysaccharide interaction in the food industry is an emerging research domain. Both polyphenols and polysaccharides exhibit diverse bioactivities. When these macromolecules interact with each other and form a complex, it can be used as a nutraceutical targeting major health complications such as diabetes, obesity, and cardiovascular diseases, among others.

Further investigation is necessary to understand the underlying structural and molecular level changes within the complex and the mechanisms involved. This knowledge will be crucial for developing innovative plant-based diets, formulations, and supplements. However, specific challenges still exist in this domain, making a complete understanding of the polyphenol-polysaccharide complex difficult. Once these drawbacks are addressed, these complexes could be more spotlighted and utilized for various applications in different domains. The needs and key drawbacks include:

• Structural characterization of the polyphenol and polysaccharide complex and their impact on the complex formation still need more research. The molecular level changes in the structural parameters can be focused on, and detailed information regarding the complex formation can be established.
• Most studies investigating the bioactivity of polyphenol–polysaccharide complexes have been limited to in vitro conditions. However, to thoroughly understand their potential in functional food development and nutraceutical applications, further in vivo studies and clinical trials are essential. Moreover, deeper investigation into the molecular mechanisms underlying these interactions and the influence of diverse food matrices on their behavior and efficacy is crucial to support their effective utilization and commercialization.
• Comparative analysis needs to be conducted among different food matrices to form the polysaccharide-polyphenol complex, which could aid in the commercial application of these complexes.
• Further research should be carried out on the stability of these complexes, as the polyphenols are unstable molecules. Enhancement of the stability of the complex could aid in product development at a commercial scale.

## Data Availability

No datasets were generated or analysed during the current study.
